# Recent synthetic efforts in the preparation of 2-(3,4)-alkenyl (aryl) quinoline molecules towards anti-kinetoplastid agents

**DOI:** 10.1039/c9ra09905k

**Published:** 2020-01-29

**Authors:** Dayana Orozco, Vladimir V. Kouznetsov, Armando Bermúdez, Leonor Y. Vargas Méndez, Arturo René Mendoza Salgado, Carlos Mario Meléndez Gómez

**Affiliations:** Grupo de Investigación en Química Orgánica y Biomédica, Programa de Química, Facultad de Ciencias Básicas, Universidad del Atlántico A.A.1890 Barranquilla Colombia carlosmelendez@mail.uniatlantico.edu.co; Laboratorio de Química Orgánica y Biomolecular, CMN, Parque Tecnológico Guatiguara, Universidad Industrial de Santander Km 2 Vía Refugio, A.A. 681011 Bucaramanga Colombia kouznet@uis.edu.co; Grupo de Investigaciones Ambientales para el Desarrollo Sostenible, Facultad de Química Ambiental, Universidad Santo Tomás A. A. 1076 Bucaramanga Colombia

## Abstract

Leishmaniasis, Chagas disease and African sleeping sickness have been considered some of the most important tropical protozoan afflictions. As the number of drugs currently available to treat these human illnesses is severely limited and the majority has poor safety profiles and complicated administration schedules, actually there is an urgent need to develop new effective, safe and cost-effective drugs. Because quinoline alkaloids with antiprotozoal activity (quinine, chimanine, cryptolepine or huperzine groups) were historically and are still essential models for drug research to combat these parasitic infections, synthetic or semi-synthetic quinoline-based molecules are important for anti-kinetoplastid drug design approaches and synthetic methods of their preparation become a key task that is the central subject of this review. Its goal is to highlight the advances in the conventional and current syntheses of new 2-(3,4)-alkenyl (aryl) quinoline derivatives, which kill the most important kinetoplastid protozoa, – *Leishmania* and *Trypanosoma* and could be useful models for antileishmanial and antitrypanosomal research. An attempt has been made to present and discuss the more recent contributions in this field over the period 2015–2019, paying special attention to molecular design, synthetic efforts to new green reaction conditions for classical methods such as Skraup synthesis, Friedländer synthesis, Conrad–Limpach, Doebner–Miller, as well as contemporary methods like Gould–Jacobs, Meth–Cohn and Povarov reactions. This review includes brief general information on these neglected tropical diseases, their current chemotherapies, and primary natural models (quinoline alkaloids), suitable for development of anti-kinetoplastid quinoline-based agents. The main part of the review comprises critical discussion on the synthesis and chemistry of new quinolines diversely substituted by alkyl (alkenyl, aryl) fragments on the pyridine part of the quinoline skeleton, which could be considered interesting analogues of chimanine alkaloids. The methods described in this review were developed with the aim of overcoming the drawbacks of the traditional protocols using revolutionary precursors and strategies.

## Abstract 2

Leishmaniasis, Chagas disease and African sleeping sickness have been considered some of the most important tropical protozoan afflictions.

## Graphical abstract

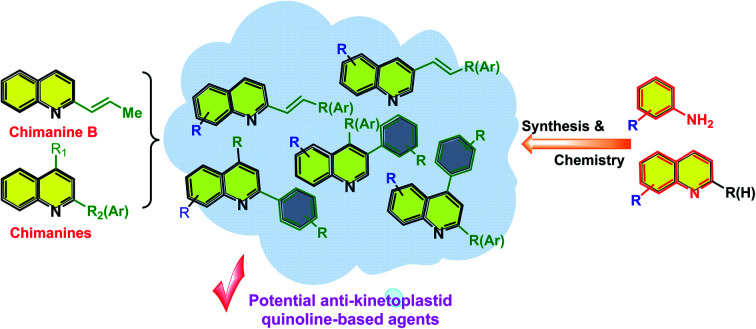

## Introduction

Among diverse classes of heterocyclic molecules, quinoline-containing compounds stand out as important molecules displaying a wide spectrum of chemical, physical and biological activities. Simple quinoline derivatives are applied in the manufacture of dyes, paints, insecticides and antifungals. They also are employed as solvents for the extraction of resins and terpenes, and as corrosion inhibitors. However, the quinoline ring is also a key structural unit for numerous natural products and privileged scaffolds in medicinal chemistry. A quinoline nucleus is generally present in a large number of synthetic and natural molecules with relevant parasite growth inhibition properties. Currently, quinoline derivatives are available as antimalarial (chloroquine, mefloquine, amodiaquinine, primaquine, *etc.*), antibacterial (ciprofloxacin, sparfoxacin, gatifloxacin, *etc.*) or anticancer drugs (camptothecin, irinotecan, topotecan, *etc.*).

However, no quinoline derivative has yet become an antileishmanial or antitrypanosomal drug that could be used in the treatment of leishmaniasis, Chagas disease or African sleeping sickness. These so-called neglected tropical diseases affect 20 million people worldwide leading to more than 100 000 deaths annually.^1–3^

Taking into consideration that quinoline-containing compounds have a pivotal role in design and development of antimalarial drugs (*i.e.*, alkaloid quinine isolated as the active ingredient from the crude back (Peruvian bark) of Cinchona trees, *Cinchona officinalis, C. pubescens*, *etc.*),^4^ there have been many efforts to develop new antileishmanial and antitrypanosomal drugs using antimalarial quinoline agents as suitable models. Indeed, the need for antimalarial drugs based on quinoline ring has accelerated the development of methods of synthesis of quinolines. It is well-known that the core of quinoline could be synthesized using diverse historical or classical named reactions such as Skraup, Doebner–von Miller, Friedländer, Pfitzinger, Conrad–Limpach, Combes syntheses, as well as Gould–Jacobs, Meth–Cohn and Povarov reactions. These classical methods for quinoline synthesis have been widely studied and frequently used for the preparation and molecular diversification of the quinoline ring system.^5^ However, each year new diverse reaction conditions modifications of these reactions are appeared. These advances are periodically analysed. As a consequence, there are many reviews and reports on this theme in the scientific literature^6–11^ that confirmed that metal- and acid-catalysed coupling cyclization or cycloaddition reactions can harmonize the classical syntheses offering higher efficacy, rapidity and a greater orientation to molecular diversification. These powerful reactions provide new antileishmanial and antitrypanosomal agents.

Selected examples of the synthesis of such promising quinoline molecules are the central subject of this review that is divided in the following two sections: a brief background and main part called antitrypanosomal quinoline-based molecules. Both sections include some divisions that help to highlight the advances in the current syntheses and chemistry of quinoline derivatives active against leishmaniasis, Chagas disease and African sleeping sickness.

## Leishmaniasis, Chagas disease and African sleeping sickness as neglected tropical diseases

**Fig. 1:**
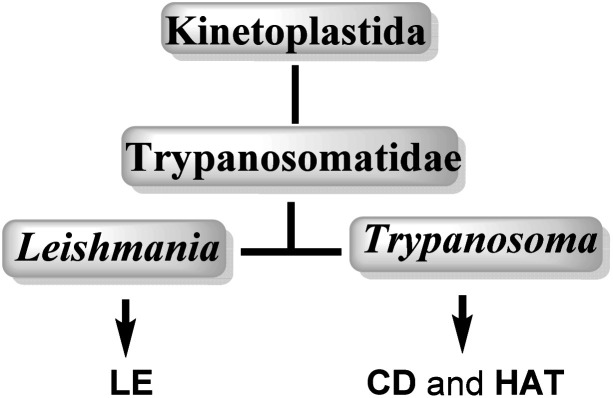
Taxonomy of medicinally important kinetoplastid protozoa.

Neglected tropical diseases (NTDs) leishmaniasis (LE), Chagas disease (CD) and African sleeping sickness or human African trypanosomiasis (HAT) are caused by a group of flagellated protozoans, trypanosomatids (Trypanosomatidae family), which belongs to flagellated protists of the kinetoplastids (Kinetoplastida order), identified by the presence of a DNA-containing region, known as a “kinetoplast,” in their single large mitochondrion. Trypanosomatids are relatively early branching eukaryotic organisms, and their cell organization is substantially different to that of mammals. The etiological agents that cause LE, CD and HAT infections, are grouped into two important genuses: *Leishmania* spp. and *Trypanosoma* spp.^12,13^ (Fig. 1, Table 1).

**Table 1:** The most important characteristics of leishmaniasis, Chagas disease and African sleeping sickness

NTDs	Causative organisms	Vectors/transmission	Global incidence	Population at risk	Principal disease forms (stages)
LE	Over 20 *Leishmania* spp.: most important *L. donovani*, *L. major*, and *L. braziliensis*	Phebotomine sandflies: *Phlebotomus* spp. and *Lutzomyia* spp./infection *via* fly bite	12 million	350 million	Main clinical forms: cutaneous, mucocutaneous and visceral
CD	*Trypanosoma cruzi*	Reduviid bugs: *Rhodnius* spp., *Triatoma* spp./infection by feces of infected buds	8–9 million	100 million	Acute phase and chronic phase
HAT	*T. brucei gambiense*, *T. brucei rhodesiense*	Tsetse fly (genus *Glossina*)/infection *via* fly bite	<1 million	50 million	Early stage (hemo-lymphatic) and late stage (CNS)

In particular, *Trypanosoma* is responsible for Chagas disease (*Trypanosoma cruzi*) in South America and sleeping sickness in sub-Saharan Africa (*Trypanosoma brucei* subsp.), whereas *Leishmania* is responsible for cutaneous and visceral infections, endemic in 88 countries mostly in the Horn of Africa, South Asia and Latin America, with cases also recorded in Spain and in the south of Italy (over 20 *Leishmania* spp.). These kinetoplastid diseases are transmitted by different insect vectors (Table 1).^14–17^

Human African trypanosomiasis (HAT) or sleeping sickness is a lethal disease, if left untreated and produces approx. 48 000 deaths annually. While the symptoms of the haemolymphatic stage are mostly nonspecific and include fever, headache and swelling of the lymph nodes, the central nervous system (CNS) is affected in the second meningoencephalitic stage producing convulsions and alteration of the circadian rhythm, a characteristic giving the disease its name.

There are two main stages of the Chagas disease: the acute stage, which appears shortly after the infection, and the chronic stage, which may last for several years and lead to chronic heart failure, digestive lesions and peripheral nervous damage. This disease generates approx. 14 000 deaths annually. The annual deaths produced by visceral leishmaniasis are about 51 000. Out of these, about 90% cases occur in India, Nepal, Bangladesh and Brazil.^1,2^

## Current drugs and their disadvantages

**Fig. 2:**
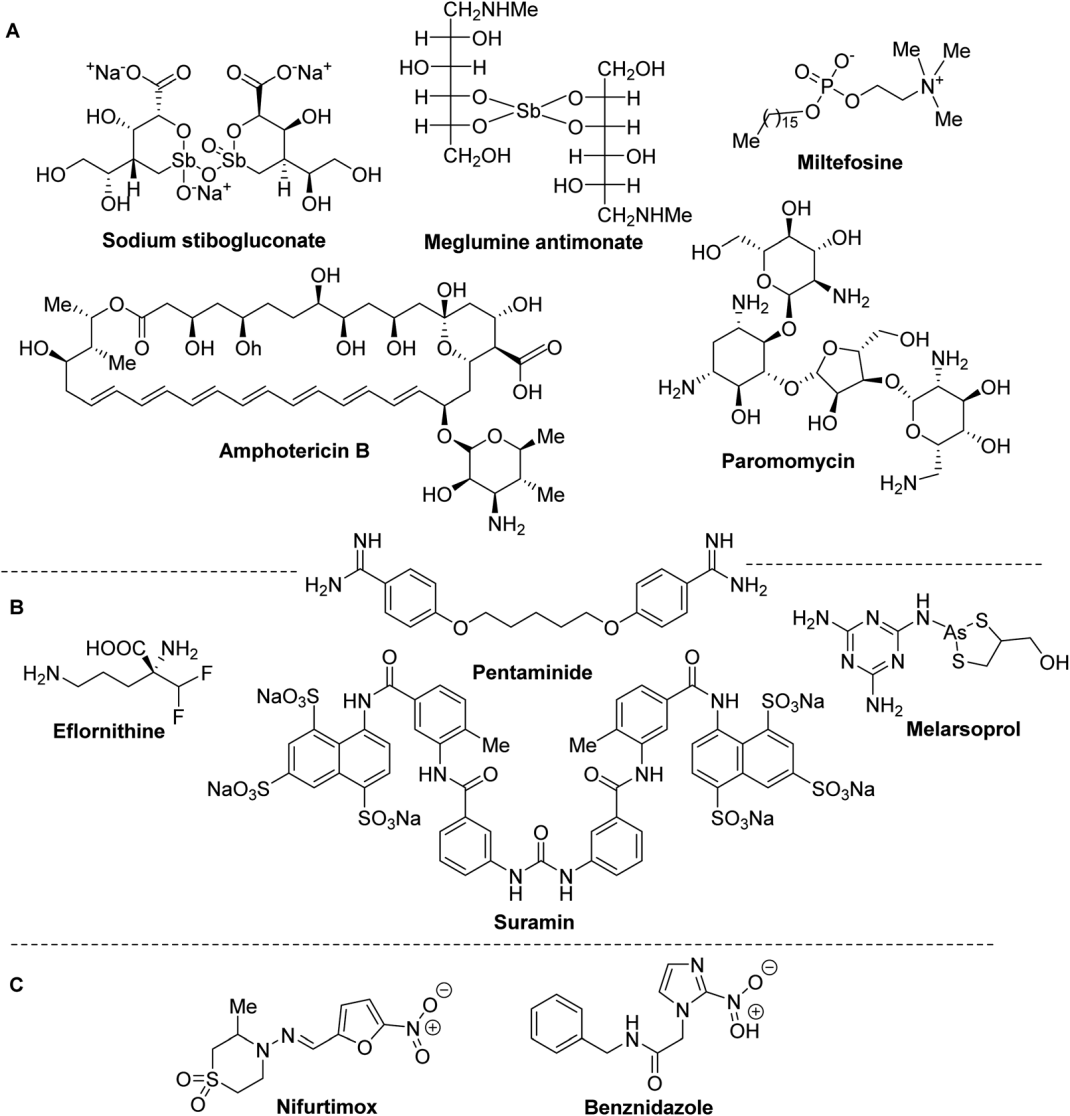
Drugs currently used in the treatment of leishmaniasis and trypanosomiasis.

Despite to the morbidity and mortality of LE, CD and HAT, until now there are very few available drugs and effective therapies. Moreover, none of these drugs (Fig. 2) and treatments based on them is ideal due to the high toxicity and severe side-effects, especially on heart, liver and kidneys, they are expensive and require repeated parental administration.

Usually, synthetic organic antimonials and arsenicals or antibiotics are used in the treatment of trypanosomiasis and leishmaniasis. Among them, sodium stibogluconate (Pentostam®) and meglumine antimoniate (Glucantime®) have been specially used since the 1930–45s as the front-line drugs of choice for all the clinical forms of leishmaniasis, while liposomal amphotericin B (AmBisome®), an antifungal antibiotic that is used with increasing frequency to treat visceral leishmaniasis. Other drugs currently used in the treatment of leishmaniasis are paromomycin, miltefosine and pentaminide (Fig. 2A).^18,19^

The latter drug is also used in therapy for human African trypanosomiasis together with the other suramin, pentaminide, melarsoprol, eflornithine^19^ (Fig. 2B). Finally, only two the nitroheteroaromatic drugs benznidazole and nifurtimox are usually in clinical use of Chagas disease (Fig. 2C).^20^

## Quinoline alkaloids as primary models drug research to fight trypanosomal infections

**Fig. 3:**
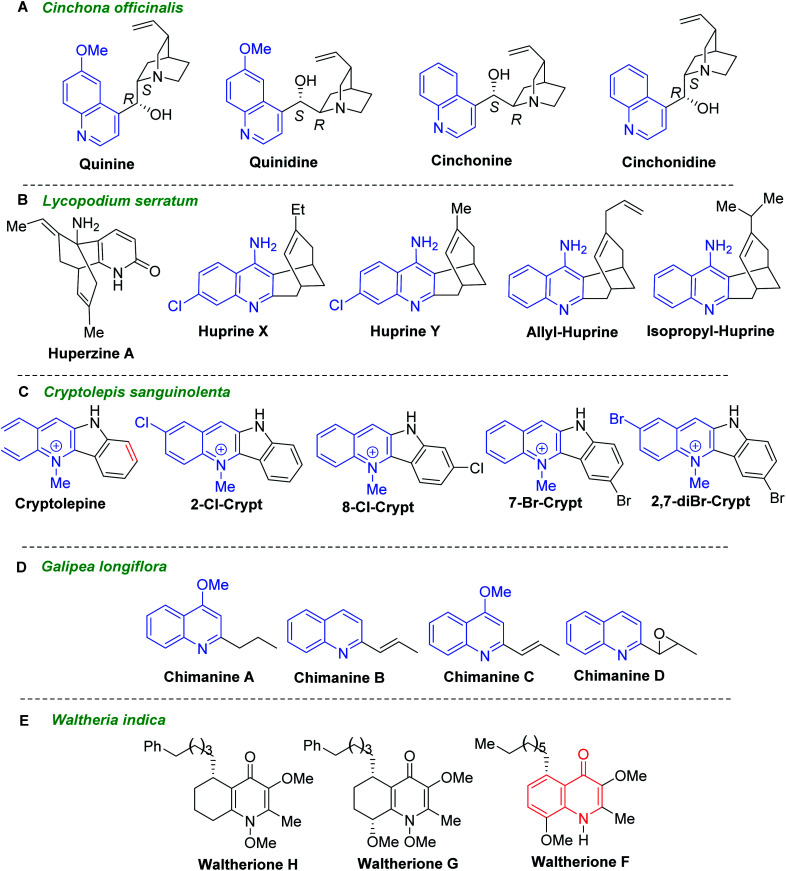
Quinoline alkaloids as model compounds for developing drugs against *Leishmania* and *Trypanosoma* parasites.

Secondary metabolites from vegetal sources, especially quinoline alkaloids provide interesting candidates for antiparasitic drugs.^21^ Historical quinoline alkaloids such as quinine, quinidine, cinchonine, and cinchonidine (Fig. 3A) are the first drugs to treat malaria came from *Cinchona officinalis* and related *Cinchona* species (Rubiaceae) which naturally occur in Central and South America. Administered as mixture, the association of quinine–quinidine–cinchonine called Quinimax® is still used in malaria therapy to treat the blood stages of *Plasmodium* parasites.^22^ The cinchona alkaloids skeleton entails two rigid rings: an aliphatic quinuclidine and an aromatic quinoline ring joined together by two carbon–carbon single bonds. These alkaloids served as a lead structure for the synthesis of several synthetic antimalarial drugs (*e.g.*, chloroquine, amodiaquine, mefloquine or primaquine) and a number of their hybrid forms.^23,24^ Noteworthy that they showed also its potent activity against *T. b. brucei* parasites with IC_50_ values below 10 μM. Having IC_50_ values of 0.8–1.2 μM and the considerable selectivity index (SI > 200, in the cell line HL-60, human promyelocytic leukemia cells), quinidine and cinchonine become potential agents for further drug development for HAT.^25,26^

Lycopodium alkaloid huperzine A, isolated from the club moss *Huperzia serrata* (Thunb., trev. = *Lycopodium serratum*), and their synthetic analogues called huprines X, Y and isopropyl- and allyl-huprines (Fig. 3B) possess a potent anti-AChE activity and exhibit also trypanocidal properties with IC_50_ values of 303–377 nM (*T. brucei*, strain 427).^27,28^ Being 4-aminoquinoline-based compounds related to antimalarial drugs, chloroquine and quinacrine, these quinoline molecules could serve for developing new antitrypanosomal agents.

Another historical antimalarial alkaloid, cryptolepine (Crytp, 5-methyl-5*H*-indolo[3,2-*b*]quinoline) from the roots of *Cryptolepis sanguinolenta* (Lindl.) Schltr. (Periplocaceae), and its synthetic halo substituted indoloquinoline derivatives (2-Cl-Crypt, 7-Br-Crypt, 8-Cl-Crypt, and 2,7-diBr-Crypt) (Fig. 3C) exhibited highly potent activity against *T. brucei* possessing IC_50_ values of less than 3 nM.^28^ Testing their cytotoxic activities against mouse adenocarcinoma of the colon cells (MAC15A), it found that 2-Cl-Crypt, 8-Cl-Crypt and 2,7-diBr-Crypt showed high selective toxicity to trypanosomes (selectivity indices, SI 8084, >3019 and 2083, respectively), while cryptolepine exhibited less antiprotozoal activity and relatively low selectivity against trypanosomes (SI = 29.3). The most effective compound against *T. brucei brucei* infection in rats was 2,7-dibromocryptolepine, those single oral dose of 20 mg kg^−1^ suppressed parasitaemia and increased the mean survival time to 13.6 days compared with 8.4 days for untreated controls.^28^

Simple 2-substituted quinoline alkaloids chimanines (Fig. 3D), isolated from leaves or trunk bark of a Bolivian medicinal plant *Galipea longiflora* Kr (Rutaceae), resulted to be interesting and promising models for the development of new antileishmanial and antitrypanosomal agents.^29,30,31^ Chimanines D and B displayed antileishmanial activity (IC_90_ = 25 μg mL^−1^) against promastigotes of *L. braziliensis*, while the 2-*n*-propylquinoline (desmethoxy analogue of chimanine A) showed activity at an IC_90_ value of 20 μg mL; it that means that they exhibited greater potency than *N*-methylglucamine antimonate against *L. amazonensis*, *L. braziliensis* and *L. donovani*.^30^ Moreover, further investigations have shown their efficacy in the experimental treatment of cutaneous leishmaniasis as well as in Balb/c mice chronically infected with *T. cruzi*, responsible for Chagas disease.

The dichloromethane root extracts of *Waltheria indica* L. (syn. *Waltheria americana*, Malvaceae) containing quinolone alkaloids showed also potent and selective growth inhibition toward *T. cruzi*. Among them, waltheriones G, H and F (Fig. 3E) stand out as remarkable antichagasic agents with IC_50_ values between 0.02 and 1.00 μM.^32,33^

From selected examples discussed above it can be concluded that on-going researches focused on natural products have shown a wise way to get a true and potentially rich source of drug candidates against these protozoan afflictions, where quinoline alkaloids play an important role as suitable models in research and development of new antitrypanosomal drugs. In order to build new active molecules based on quinoline skeletons, synthetic routes to obtain these heterocycles must be developed, improved and invented. Actually, there are many different reviews and reports on this theme in the scientific literature.^5–10^ But, our work aims to discuss selected examples of synthesis of the most active and promising antitrypanosomal quinoline-based molecules paying attention on the particular role of initial quinoline ring construction based on classical named reactions, which allow obtaining an additional value of starting quinoline precursors.

## Classical and contemporary approaches to quinolines synthesis

**Scheme 1:**
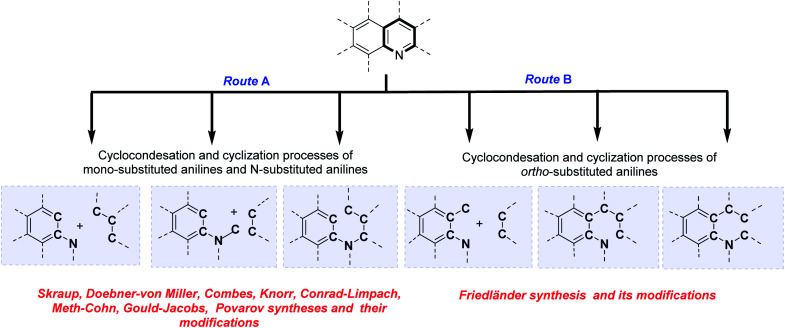
Classical and contemporary approaches to quinolines synthesis.

The analysis of the classical and contemporary approaches used in the quinoline core construction suggests two general synthetic routes: (a) the utilization of mono-substituted anilines and *N*-substituted anilines and (b) the use of *ortho*-substituted anilines. Both routes use cyclocondensation and cyclization processes (Scheme 1). Particularly interesting seems the approach employing aromatic primary amines as the nucleophilic nitrogen donating component as C_Ar_–C_Ar_–N fragment and electrophilic C–C–C three-carbon unit (usually carbonyl compounds) or C_Ar_–C_Ar_–N–C fragment and two-carbon C–C unit, referring to historical named reactions like Skraup, Doebner–von Miller, Combes, Knorr, Conrad–Limpach or contemporary Gould–Jacobs, Meth–Cohn, and Povarov reactions. The *N*-alkynyl (alkenyl)anilines cyclization reactions (C_Ar_–C_Ar_–N–C–C–C fragment) are also form part of this group (Scheme 1, route A).

Second common method of quinoline construction utilizes *ortho*-substituted anilines with the C–C_Ar_–C_Ar_–N unit and two-carbon C–C unit, usually carbonyl compounds containing a reactive α-methylene group, where Friedländer synthesis and its modifications stand out as the most important and popular methods for the construction of polyfunctionalized quinolines. Other skeletons like C–C–C–C_Ar_–C_Ar_–N or C–C–C_Ar_–N–C–C are also exploited as unique components in the quinoline synthesis (Scheme 1, route B).^6,9^

These classical methods for quinoline synthesis have been widely studied and frequently used for the preparation and molecular diversification of the quinoline ring system. However, many of these procedures suffer from various problems such as low yields, harsh reaction conditions, difficulties in workup, expensive reagents, long reaction times, and the use of toxic solvents.

On the other hand, following pharmaceutical green chemistry principles, which seek to eliminate unnecessary environmental impact, but deliver efficiently life-saving medicines, each synthesis including quinoline synthesis, must strive for the right choice of starting material, ideal number and order of chemical steps, the suitable use of solvents and reagents, and effective strategies for isolation and purification.^34,35^ Moreover, sometimes there are not available green methods for the preparation of active antitrypanosomal quinoline molecules, but in general, the developments in the quinoline chemistry towards new antiparasitic quinoline agents must harmonize the classical syntheses offering higher efficacy, promptness and a greater orientation to molecular diversification.

## Synthesis and chemistry of quinolines for antiparasitic quinoline-based agents

**Fig. 4:**
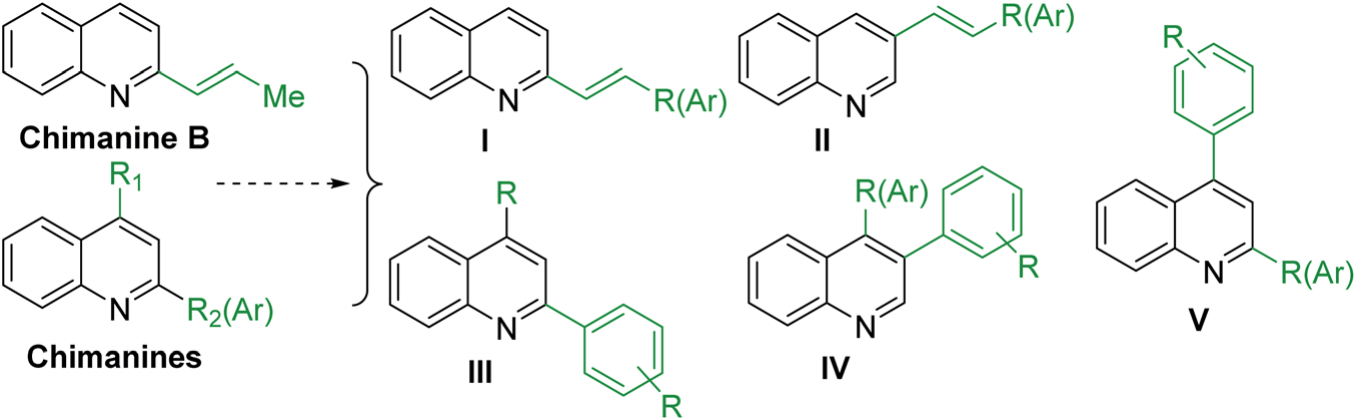
Functional diversity of quinoline ring generated using chimanines (especially, chimanine B) alkaloid models.

As the scientific interests for antitrypanosomal quinoline-based molecules started with the discovery of chimanine alkaloids, – C-2 alkyl (alkenyl) quinolines (Fig. 3D), our discussion is mainly based on the recent syntheses and chemistry of diverse quinolines alkyl (alkenyl, aryl) substituted on pyridine ring of quinoline skeleton and related quinoline derivatives with potential biological properties against the most important kinetoplastid protozoa *Leishmania* and *Trypanosoma* (Fig. 4).

**Scheme 2:**
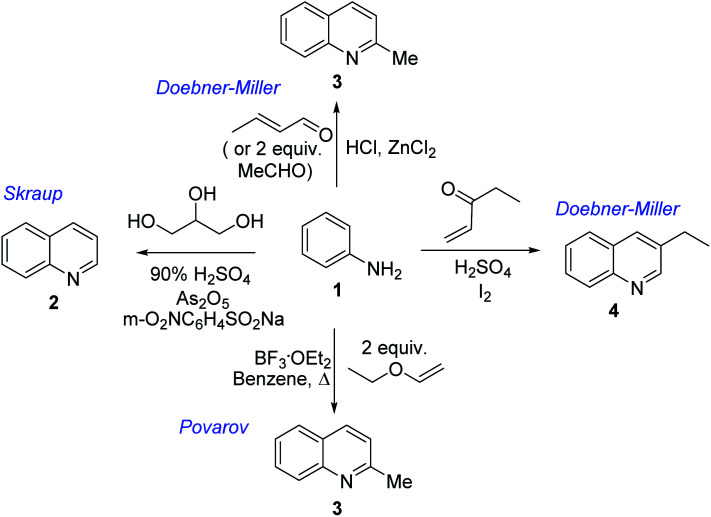
Preparation of initial quinoline precursors by Skraup–Doebner–Miller methods and Povarov reaction.

Chimanine alkaloids models, especially chimanine B [2-(*E*)-prop-1′-enylquinoline], 2-*n*-propylquinoline and 2-phenylquinoline have been used in order to find some quinoline structure-antitrypanosomal activity relations and more than 300 mono-substituted quinoline compounds of types I–IV have been early prepared and tested *in vitro* and *in vivo* against various *Leishmania* and/or *Trypanosoma* species (Fig. 4).^36–39^ Among these 2-alkenyl-(I), 3-alkenyl-(II), 2-aryl-(III), 3-aryl-(IV) and 4-aryl-(V) quinoline derivatives, several synthetic analogues demonstrated a better biological behaviour inhibiting significantly *L. infantum*, *L. donovani* or *L. amazoniensis* amastigote proliferation in macrophages.^40^ As all these molecules are simple 2- or 3-substituted quinolines and their preparation are mainly based on the Skraup–Doebner–Miller synthesis^41^ as well as the Povarov reaction.^42^ Such syntheses have currently found widespread use. The construction of the quinoline system by the Skraup–Doebner–Miller methods and the Povarov reaction is generally based on the reaction of an aromatic amine 1 containing at least one free *ortho* position with a reagent providing a source of the three-carbon C–C–C fragment such as chemical equivalents glycerol (Skraup, 1880), α,β-unsaturated aldehydes (Doebner–Miller, 1883) or formally, two-carbon C–C unit like α,β-unsaturated ethers (AB^2^ type Povarov, 1964). Classical form of the Skraup reaction (the reaction of substituted anilines with glycerol) allows obtaining only quinolines unsubstituted in the pyridine ring, while the use of α,β-unsaturated aldehydes such as crotonaldehyde, 2-ethyl acrolein or vinyl ether leads to the formation of quinolines substituted in the pyridine ring with wholly satisfactory yields. Thus, simple quinoline 2, 2-methyl- or 3-ethylquinolines 3, 4 are available by these methods (Scheme 2) and their traditional chemical transformations lead to the quinoline of types I–IV. The modified Skraup quinoline synthesis (refluxing anilines in 6 M HCl and crotonaldehyde in toluene) is still used to prepare non-commercially available 2-quinaldines.^43^

**Scheme 3:**
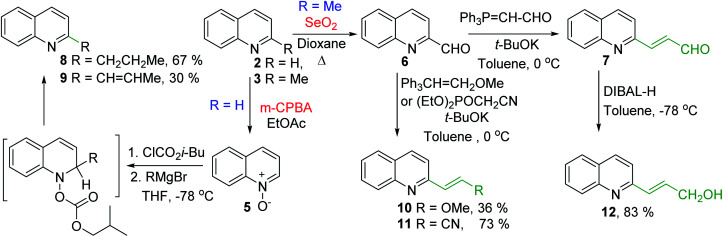
Synthesis of potent antileishmanial quinoline agents 8–12.

Some of the promising antileishmanial quinoline agents, such as 2-*n*-propylquinoline 8, (*E*)-2-(prop-1-en-1-yl)quinoline 9, 2-(2-methoxyethenyl)quinoline 10, quinolin-2-yl-acrylonitrile 11 and 2-(2-hydroxyprop-2-enyl)quinoline 12^38,44–46^ have been easily prepared from quinoline 2 or 2-methylquinoline 3 using reactions of quinoline-*N*-oxide 5 activated by iso-butyl chloroformate and Grignard reagents or Wittig reactions of 2-quinaldehyde 6 and respective Wittig reagents^36,37,47,48^ (Scheme 3).

Interestingly, 2-*n*-propylquinoline 8, a simple quinoline and available synthetic product, is the major alkaloid isolated from *G. longiflora*.^29^ This quinoline is not active *in vitro* experiments (IC_50_ > 100 μM), however its *in vivo* action (*L. donovani*/Balb/C mice model) results to be significant showing activity more effective than that of chimanine B alkaloid (quinoline 9) and antileishmanial drug meglumine antimoniate.^47^ Regarding synthetic chimanine B analogues 10–12, all these exhibited potent *in vitro* activity (IC_50_ = 2.4–3.6 μM) against resistant strains *L. donovani* promastigotes. Noteworthy, 2-alkenylquinolines 10 and 12 were found approximately 3 times more active than miltefosine^38^ and quinoline 11 was tested *in vivo L. donovani*/Balb/C mice model producing a significant reduction of parasite burden in liver in *L. donovani* infected mice (68.9%; *P* < 0.002) at 12.5 mg kg^−1^ for 10 days. This efficacy was similar to the oral treatment with the reference drug, meltifosine at 7.5 mg kg^−1^ for 10 days (72.5%; *P* < 0.001).^46^

Nevertheless, the final selection of a potential drug candidate among these quinolines 8–12 was generally based on chemical stability and acute oral toxicity as discriminating criteria and the most stable and more suitable compound in various conditions resulted to be 2-*n*-propylquinoline 8. Thus, this simple quinoline is currently a drug-candidate for the treatment of visceral leishmaniasis in pre-clinical development.^45^ As this compound is in an oily state, it was developed some formulations (as camphorsulfonic salt and hydroxypropyl beta-cyclodextrin/2-*n*-propylquinoline mixture) that are compatible with an intravenous administration and did not alter the activity of the active ingredient.^45,46^

One serious shortcoming of these reactions is the laborious procedure for the isolation of the quinoline derivatives from the reaction mixture. This is due to parallel polymerization of the α,β-unsaturated aldehydes catalysed by the acid and results in low yields. Further transformations are also rather difficult and involve the use of toxic reagents and solvents. Investigations of recent years have therefore been devoted to the search for better conditions for the reactions.

The use of microwave (MW) heating in place of conventional heat sources demonstrated to be an efficient method for the synthesis of quinoline derivatives like 2, 3 exploiting the Skraup reaction of anilines 1 and glycerol.^49^ However, these procedures utilize corrosive H_2_SO_4_. Similar continuous flow Skraup reaction of solketal and anilines over a solid acid niobium phosphate (NbP) instead of corrosive sulphuric acid gave selectivity for quinoline of up to 60%.^50^ Diverse green reaction media like 1-(1-alkylsulfonic)-3-methylimidazolium chloride Brønsted acidic ionic liquids have shown as excellent catalysts and reaction mediums for Skraup synthesis of quinolines under MW heating without the use of nitrobenzene as an oxidant and metal catalysts. This protocol allowed obtaining the quinolines in good yields (67–76%).^51^

However, innovative conditions for efficient quinoline preparation throughout Skraup–Doebner–Miller reactions are still needed. A novel and green route for vapour-phase synthesis of 2- and 4-methylquinolines (a 67.6% total yield of quinolines) *via* modified Doebner–Miller reaction starting from aniline and lactic acid in the presence of HBeta zeolite catalysts was reported recently.^52^ This reaction process consists of decarbonylation of lactic acid to acetaldehyde and subsequent condensation of acetaldehyde and aniline to 2- and 4-methylquinolines. Samiei and co-workers developed also simple efficient procedure for one-pot preparation of polyfunctionalized 2-methylquinolines using a mixture of anilines and acetaldehyde (in excess) on Al_2_O_3_/HCl under MW irradiation (5–10 min) without any solvents.^53^

**Scheme 4:**
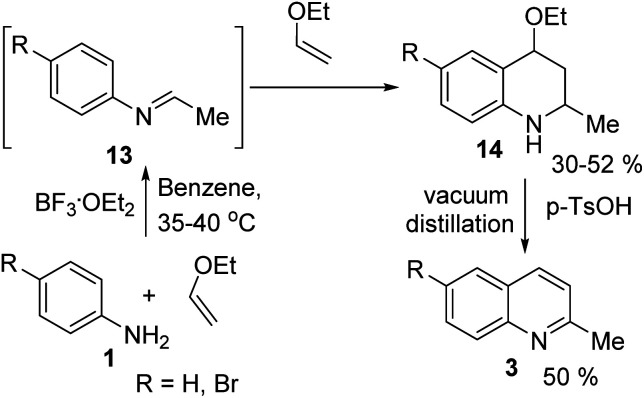
First example of quinaldine synthesis by Povarov procedure.

However, the Povarov reaction of anilines and ethyl vinyl ether (as an aldehyde surrogate) in the presence of acid catalysts could be considered a better alternative approach to the 2-methyquinolines Doebner–Miller synthesis. The original version of this transformation was developed by Povarov and Mikhailov in 1964 and consisted in the initial BF_3_·OEt_2_-promoted reaction of anilines 1 and ethyl vinyl ether in benzene to form *N*-methyl aldimines 13 and subsequently underwent an imino Diels–Alder reaction with a second molecule of ethyl vinyl ether to afford the corresponding 2-methyl-4-ethoxy-1,2,3,4-tetrahydroquinolines 14 which were converted into quinaldine derivatives 3 under vacuum distillation conditions in the presence of *p*-toluenesulfonic acid (*p*-TsOH) (Scheme 4).^42^ Nowadays, this protocol was considerably improved using various regimes of the reaction conditions (Table 2).

**Table 2:** Synthesis of substituted 2-methylquinolines under different reaction conditions: selected examples

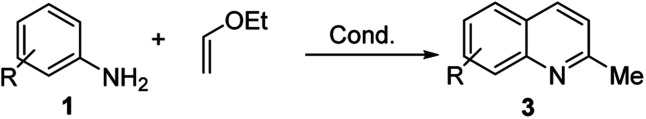			
2-Methylquinoline products 3	Yield (%) and reaction conditions		
	5 mol% PdCl_2_/MeCN	AcOH	5 mol% I_2_/C_6_H_6_
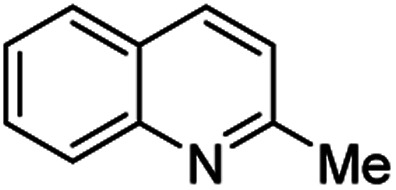	82	72	37
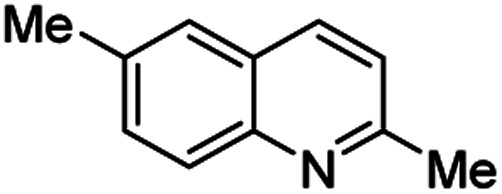	82	60	53
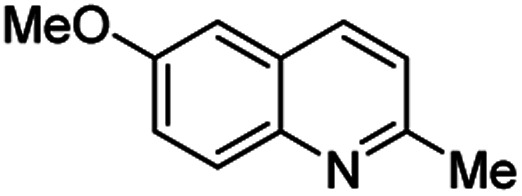	86	78	64
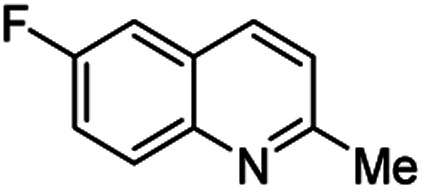	77	68	nt
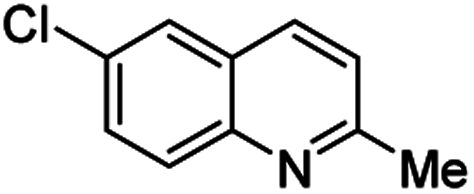	70	68	nt
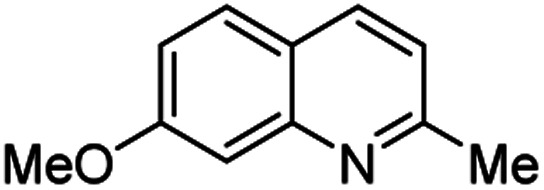	62	75	30
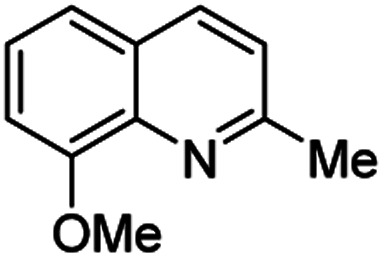	74	nt^a^	36

a nt – not tested under these reaction conditions.

Matsubara and co-workers reported a highly efficient action of PdCl_2_ as catalyst (5 mol%) in this reaction performed in acetonitrile at 80 °C for 24–48 h. The efficiency and functional-group tolerance of this procedure have been fully demonstrated by synthesizing a number of substituted 2-methylquinolines like 3.^54^ Stirring anilines and ethyl vinyl ether (1 : 3 mole ratios) in the presence of acetic acid at room temperature for 3–4 h followed by reflux for 3.5–4 h allowed obtaining substituted 2-methylquinolines in good yield.^55^ Cheap raw materials and milder experimental conditions make this procedure an attractive option for their rapid synthesis of these quinoline derivatives.

Recently, an iodine-mediated reaction was applied to the synthesis of these quinolines. Nishiwaki and co-workers developed a mild and inexpensive method for the synthesis of 2-methylquinolines by condensation of anilines with vinyl ethers in the presence of catalytic amount of iodine (5 mol%) in benzene at 80 °C for 2 h.^56^ Interestingly, employing *p*-TsOH this reaction instead of iodine, it was demonstrated that *p*-toluenesulfonic acid did not catalyse this reaction at 80 °C and it was necessary to heat at 120 °C. In this reaction, the iodine species was revealed to show dual behaviour; molecular iodine serves as an oxidant, while its reduced form, hydrogen iodide, activates the vinyl ether.

**Scheme 5:**
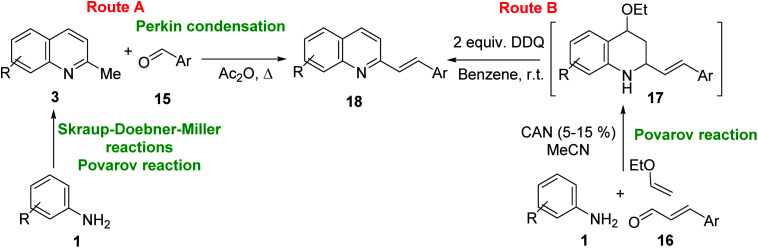
Synthetic methodologies for synthesis of 2-styrylquinolines.

One of the most interesting synthetic applications of the 2-methylquinoline derivatives consists of the use as precursors in the synthesis of (*E*)-2-styrylquinolines 18. Their traditional preparation involves the Perkin condensation reaction of 2-methylquinolines like 3 with aromatic aldehydes 15 in acetic anhydride under reflux (140 °C) for a prolonged period (∼16 h)^57–59^ (Scheme 5, route A) or the CAN-catalysed Povarov reaction between anilines 1, cinnamaldehydes 16, and vinyl ethers to form the intermediate tetrahydroquinolines 17 (like 14) followed by DDQ-promoted dehydrogenative aromatization (Scheme 5, route B).^60^

**Fig. 5:**
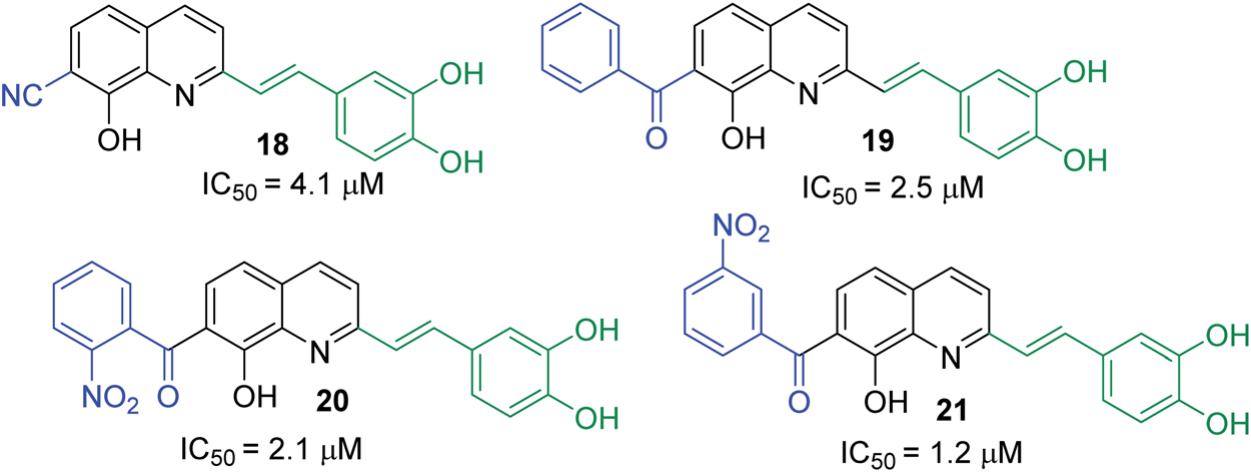
Structures of 2-styrylquinolines, promising antileishmanial agents (IC_50_, μM, *Leishmania donovani* amastigotes).

First approach has been commonly used in the preparation of antileishmanial 2-styrylquinolines. Among diverse 2-styrylquinolines, the derivatives 18–21 stand out as the most promising molecules against *L. donovani* intramacrophage amastigotes with IC_50_ values 1.2–4.1 μM (Fig. 5). 2-Styrylquinolines 18 and 21 not only displayed potent antileishmanial activity, but exhibited less cytotoxicity (KB cells) presenting a selectivity index of values 8.3 and 121.5. The latter compound resulted be 10-fold more active than miltefosine, the reference compound, with selectivity index 607-fold higher. With these parameters, it was selected as a candidate for evaluation *in vivo* with *L. donovani* mouse or hamster models.^61^

**Scheme 6:**
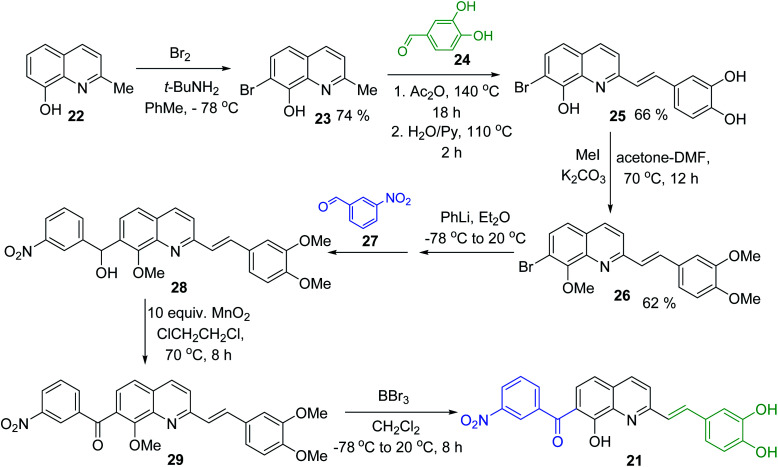
Synthesis of drug candidate 21 from 2-hydroxyquinaldine 22.

Simple synthesis of drug candidate 21 starts with readily available 8-hydroxyquinaldine 22 (Scheme 6). Its gentle bromination reaction followed by Pearson protocol (Br_2_, *t*-BuNH_2_, toluene, −78 °C)^62^ afforded 7-bromo-quinoline derivative 23. Its Perkin condensation reaction with 3,4-dihydroxybenzaldehyde 24 using classical reaction conditions (refluxing Ac_2_O, then H_2_O, pyridine, 100 °C) allowed obtaining 2-styrylquinoline derivative 25 in 66% yield. Before completing construction of this drug candidate, quinoline 25 was converted into trimethoxy derivative 26, which was submitted to Br/Li exchange according to the Quéguiner procedure (PhLi, Et_2_O, −78 °C)^63^ and the resulting lithio derivative was condensed with 3-nitrobenzaldehyde 27 giving respective quinoline alcohol 28. This alcohol was further oxidized (MnO_2_) into corresponding ketone 29 with a 50–90% yield. Finally, its treatment with BBr_3_ in CH_2_Cl_2_ at 20 °C delivered antileishmanial agent 21 (Scheme 6).^64^

Due to the importance of 2-alkenylquinolines such as 8–12 and 18–21, considerable efforts have been devoted to developing various methods to construct such motifs. As it was mentioned above, the Wittig reaction was applicable using 2-quinolinecarboxaldehyde 6 as a substrate and Wittig reagents to give 8–12 (see, Scheme 5) or 2-bromo(chloro)quinoline derivatives, Wittig reagents, and aldehydes.^65^ However, formation of a stoichiometric amount of phosphane oxide by-product often complicated the purification process. The major drawbacks of well-established strategy for synthesis of 18–21, based on the Perkin condensation reaction of 2-methylquinolines and aldehydes, are long reaction time and severe reaction conditions.

However, it should be noted that efficient microwave-assisted synthesis for diverse styrylquinolines based on the Perkin condensation reaction under solvent-free conditions have been reported.^66–71^ This method is not only faster but also improves the yields and constitutes a simple and environmentally friendly alternative for the known procedure. On the other hand, the use of transition metal catalyst in this reaction improved also considerably the yields of this reaction. In this context, synthesis of 2-alkenylquinolines *via* C(sp^3^)–H functionalization of 2-methylquinolines with aldehydes in the presence of Lewis or Brøsnted acid acids is a promising alternative for the preparation of related quinoline-based antileishmanial agents.

Thus, nowadays acid-catalysed reactions of 2-methylquinolines and aldehydes have become very popular. Wang group reported La(Pfb)_3_-catalysed reaction of 2-methylquinolines 3 and benzaldehydes 15 in boiling toluene (120 °C, air, 24 h) to prepare styrylquinolines 18 in good to excellent yields (55–99%),^72^ while Teo and co-workers described catalytic InCl_3_ (10 mol%) and CoCl_2_ (10 mol%) activation (THF, 120 °C, 24 h) of this type of reaction^73,74^ and Yaragorla group realized this reaction heating them in closed vessel at 130 °C for 4–5 h in the presence of Ca(OTf)_2_ (5 mol%) and Bu_4_NPF_6_ (2 mol%).^75^ Russian researchers also reported the synthesis of 2-styrylquinoline derivatives 18 under microwave irradiation in the presence of zinc chloride (Table 3).^76^ The developed procedure is advantageous due to shorter reaction time (5–10 min) and simpler workup.

**Table 3:** Synthesis of substituted 2-styrylquinolines under different reaction conditions: selected examples

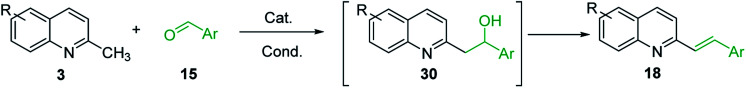				
2-Styrylquinolines 18, R = H	Yield (%) and reaction conditions			
	La(Pfb)_3_/toluene	InCl_3_/THF	Ca(OTf)_2_/Bu_4_NPF_6_	ZnCl_2_/MW
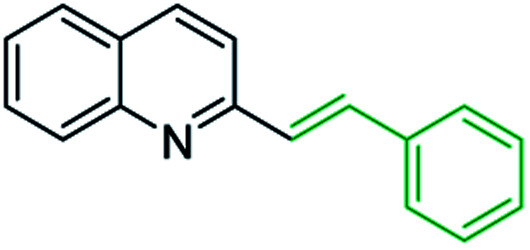	88	80	96	63
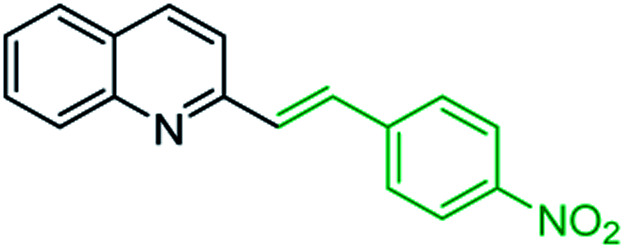	99	nt^a^	98	78
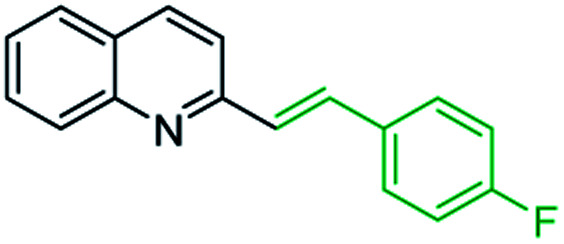	83	80	88	64
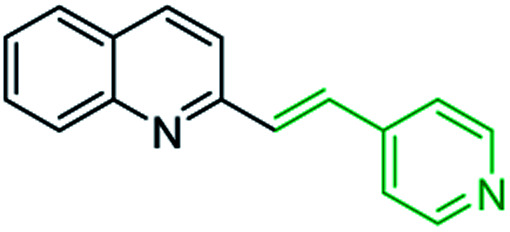	nt	80	nt	60

a nt – not tested under these reaction conditions.

**Scheme 7:**
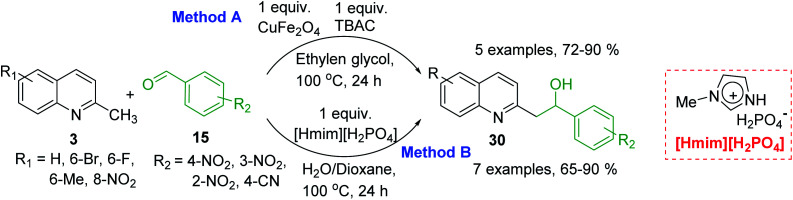
Synthesis of (quinolin-2-yl)ethan-1-ol derivatives 30.

However, it should be noted that reactions of 2-methylquinolines 3 and benzaldehydes 15 to give styrylquinoline derivatives 18 can be realized under catalyst-free condition in boiling 1,4-dioxane,^77^ while the same reactions in water under microwave irradiation furnished intermediate (quinolin-2-yl)ethan-1-ol derivatives 30.^78^ Interestingly, similar derivatives have been obtained through the copper ferrite (CuFe_2_O_4_) nanoparticle-catalysed direct C(sp^3^)–H bond functionalization of 2-methylquinolines with benzaldehydes 15^79^ (Scheme 7, method A), this functionalization can be also realized in the presence of acid ionic liquid, [Hmim][H_2_PO_4_] in water-dioxane mixture (Scheme 7, method B).^80^ The application of magnetic copper ferrite nanoparticles CuFe_2_O_4_ for the synthesis of the 2-styrylquinoline derivatives *via* C(sp^3^)–H bond activation looks particularly advantageous due to easy, quick, and quantitative separation of this catalyst from the reaction medium. This catalyst as well as acid ionic liquid [Hmim][H_2_PO_4_] can be recycled and reused for several times without significant decrease in activity.

**Scheme 8:**
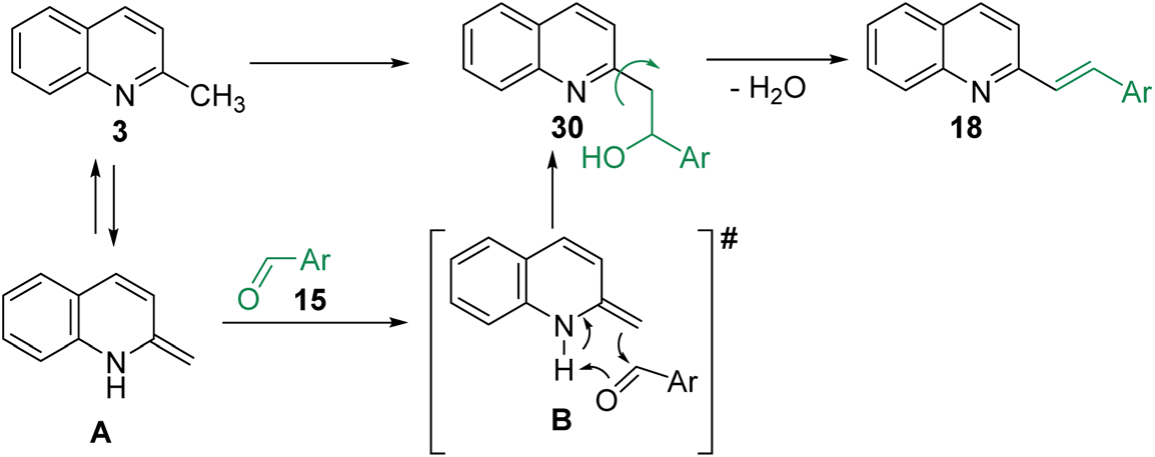
Plausible mechanism for sp^3^ functionalization of 2-methylquinolines.

The formation of Perkin condensation products through C(sp^3^)-H bond activation might be explained as follows: the enamine intermediate A generated from quinoline 3*via* tautomerization in presence of acid catalyst (or without it) attacks the carbonyl group of aromatic aldehyde 15 through quasi cyclic transition state B with hydrogen bonding, that furnishes intermediate quinolinylethan-1-ol 30, which suffers subsequent dehydration and the (*E*)-configured styrylquinoline 18 is finally afforded (Scheme 8).

**Scheme 9:**
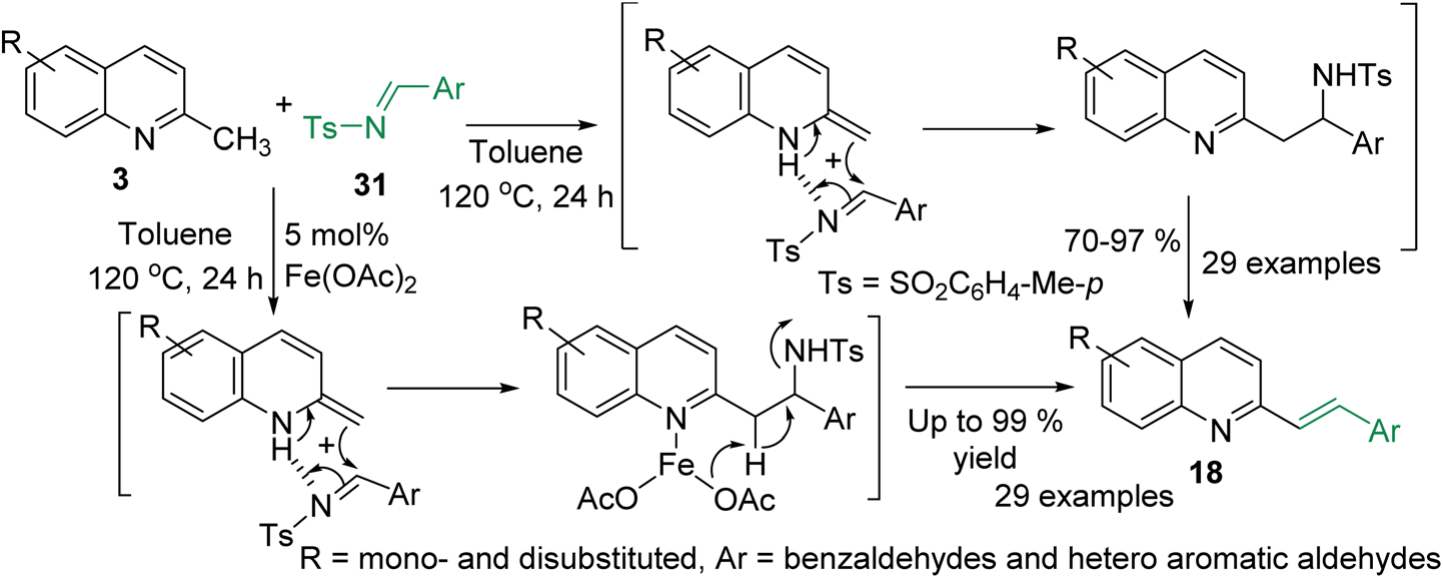
C–H functionalization of 2-methylquinolines 3 with *N*-sulfonyl aldimines.

Huang group and Wang group demonstrated simultaneously^81,82^ the viability of the synthesis of styrylquinoline derivatives *via* direct C–H functionalization of 2-methylquinolines 3 with readily accessible *N*-sulfonyl aldimines 31. Interestingly, these reactions occur well in the presence of Fe(OAc)_2_ catalyst^81^ and under catalyst-free conditions^82^ suggesting similar mechanistic route (Scheme 9).

**Scheme 10:**
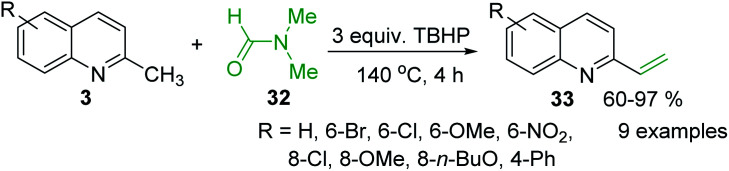
Simple and efficient synthesis of 2-vinylquinolines.

The use of *N*-aryl imines or *in situ* generated *N*-aryl imines as alternatives to aldehydes and *N*-sulfonyl imines in the process of olefination with 2-methylquinolines was also reported.^83,84^ An interesting, simple and efficient method for the synthesis of 2-vinylquinolines from of 2-methylquinolines *via* an iron-catalysed C(sp^3^)–H functionalization was recently developed by Wang and co-workers.^85^ They used the *N*,*N*-dimethylformamide 32 as false formaldehyde in the presence of *tert*-butyl hydroperoxide (TBHP) (140 °C, 4 h, under air) (Scheme 10). This simple procedure that avoids the use of organometallic compounds can provide a variety of 2-vinylquinolines 33 from readily available 2-methylquinolines 3 with good yields.

**Scheme 11:**
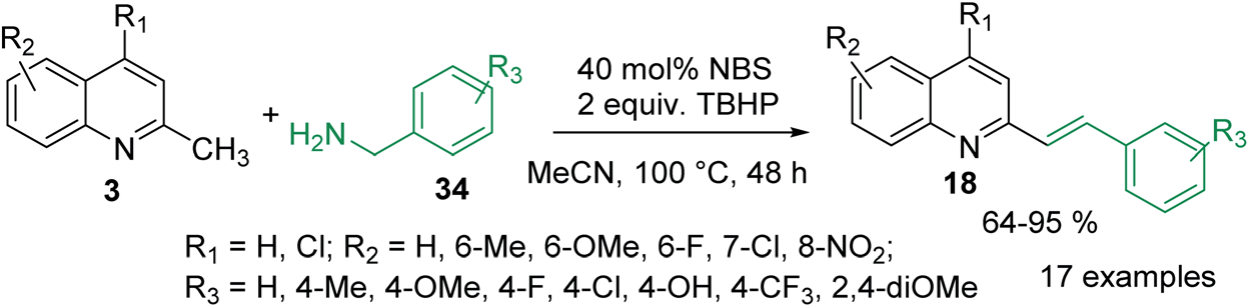
C–H functionalization of 2-methylquinolines 3 with benzylamines.

It was reported that metal-free olefination reaction between 2-methylquinolines 3 and benzylamines 34 in the presence of NBS as catalyst and TBHP as oxidant in MeCN gave 2-styrylquinolines 18 in good yields (Scheme 11).^86^ It was also demonstrated that heterocyclic methanamines, such as 2-thiophen-methanamine and 2-furanmethanamine, could be transformed into their corresponding olefination products in good yield. The authors suggested that under established reaction condition benzylamine could convert into benzaldehyde, which further reacts with 2-methylquinoline *via* aldol-type condensation to give the olefination product 18. Similar 2-styrylquinoline molecules can be prepared through microwave-assisted Sonogashira coupling between 2-iodoanilines and 4-*N*,*N*-dimethylamino-2-*trans*-styryl propargyl alcohol.^87^ Diverse 2-styrylquinolines are also known antiviral and anticancer agents.^88–90^

**Scheme 12:**
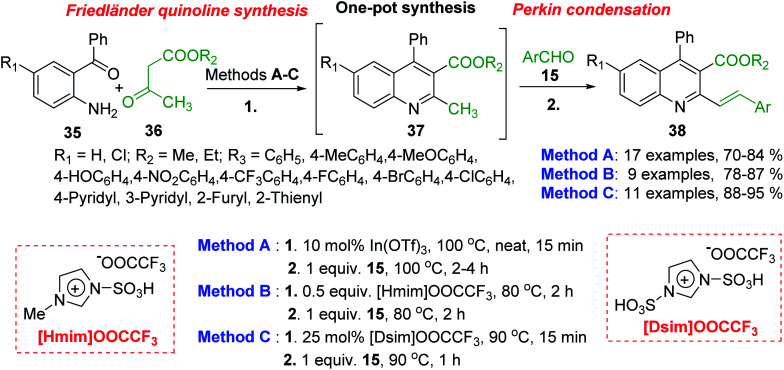
One-pot procedure for the synthesis of 2-styrylquinolines.

Polyfunctionalized 2-styrylquinolines 38 are now easily available through the tandem Friedländer annulation-Knoevenagel (Perkin) condensation involving 2-aminobenzophenones 35, β-keto esters 36 (methyl or ethyl acetoacetates) and benzaldehydes 15. This transformation occurs under solvent free condition and in the presence of Lewis acids (In(OTf)_3_),^91^ acid ionic liquids such as 1-methylimidazolium trifluoroacetate, [Hmim][OOCCF_3_],^92^ or the SO_3_H-functionalized ionic liquids, so-called task-specific Brønsted acidic liquids like 1,3-disulfo-imidazolium trifluoroacetate, ([Dsim][OOCCF_3_]) (Scheme 12).^93^

All three protocols (methods A–B) are compatible with different variation of aromatic/hetero-aromatic aldehydes giving highly functionalized 2-styrylquinolines. The catalyst, In(OTf)_3_ and the media reaction, acid ionic liquids can be recovered and reused to afford the desired products. However, according to green chemistry principles, these latter methodologies are cost effective and amenable to large scale synthesis.

**Scheme 13:**
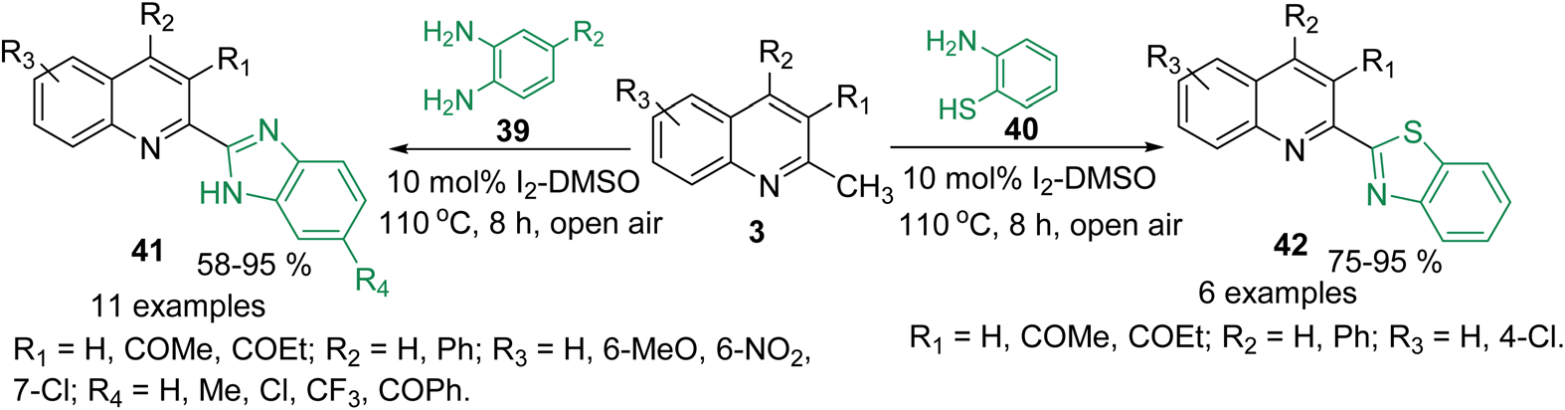
Synthesis of new quinoline hybrids from methylquinolines *via* oxidative C(sp^3^)–H functionalization process.

Oxidative C(sp^3^)–H functionalization of 2-methylquinolines could provide new potential antitrypanosomal 2-heteryl substituted quinolines such as quinoline-benzimidazole and -benzothiazole hybrids 41 and 42, which were obtained using condensation reaction between quinolines 3 and *ortho*-phenylenediamines 39 and 2-aminobenzenethiol 40, respectively. Such condensations were possible using simple I_2_-DMSO oxidative system in open flask (Scheme 13).^94,95^ These potential bioactive hybrids were prepared in high yields with good substrate scope and functional group tolerance.

Analogously, synthesis of 3-(quinolin-2-yl)isoxazoles through a 1,3-dipolar cycloaddition of 2-methylquinoline, *tert*-butyl nitrite (*tert*-BuONO) and terminal phenylacetylenes has been recently developed.^96^ In this method, nitrite serves as a suitable N–O source to transform 2-methylquinoline into intermediate nitrile oxides *in situ*.

**Scheme 14:**
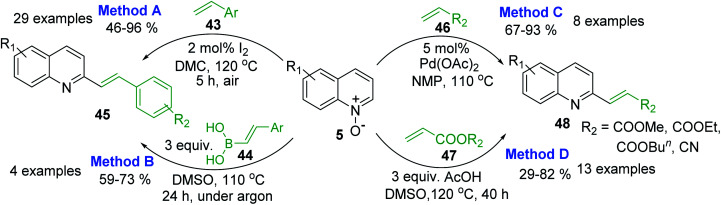
Direct C–H alkenylation of quinoline *N*-oxides increases molecular quinoline diversity.

However, 2-methylquinolines are not unique substrates to construct rapidly new 2-alkenylquinolines. Direct C–H alkenylation of quinoline *N*-oxides 5 with styrenes 43 in the presence of iodine under air atmosphere seems to be more suitable procedure for the preparation of quinoline molecules such as 45 (Scheme 14, method A).^97^ This metal-free procedure, which is really mild, simple, and efficient, has many advantages than another metal-free reaction involving arylboronic acids 44^98^ (Scheme 19, method B) or Pd-catalysed reactions of 5 and acrylates 46, 47 to give 2-alkenylquinolines 48^99^ (Scheme 14, method C) as well as other metal-free process involving simple AcOH as additive (Scheme 19, method D).^100^ Noteworthy, these metal-free reactions could be performed on a gram scale and allow to obtain easily potent antileishmanial agents such as quinolines 11 and 12, whose syntheses require several steps starting from various substituted anilines or are not in accordance with the principles of green chemistry.

Considering that quinoline *N*-oxides are more abundant and obtainable (through a simple *N*-oxidation reaction of the respective quinolines) than 2-quinolinecarboxaldehydes or 2-methyl-quinolines, direct alkenylation of quinolines at 2-positions are very attractive and enrich the structural diversity of the quinoline moiety of 2-substituted quinoline that is particularly significant in the medicinal chemistry of antiparasitic agents and the pharmaceutical industry. Thus, it is not surprising that such functionalization of quinoline *N*-oxides and related heterocycles *via* C–H bond activation is a dynamic field.^101^

**Scheme 15:**
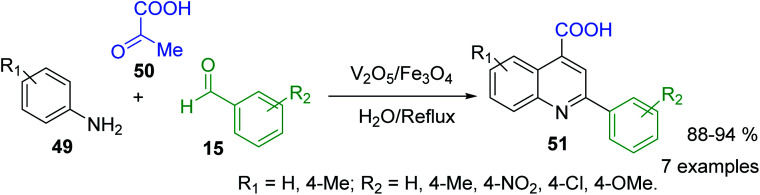
V_2_O_5_/Fe_3_O_4_-catalysed Doebner reaction.

Because of the diverse pharmacological value of 2-arylquinolines, considerable efforts have been also made to develop various synthetic methods for the synthesis of the substituted aryl quinolines. However, Doebner reaction, which involves in the interaction of aniline, aldehyde and pyruvic acid to obtain 2-substituted quinoline-4-carboxylic acids, was really the first three component reaction (3-CR), type ABC for the preparation of substituted quinolines. Nowadays, its diverse modifications correspond to the use of new reactants, catalysts and conditions. For example, V_2_O_5_/Fe_3_O_4_-catalysed Doebner reaction between diverse arylamines 49, benzaldehydes 15 and pyruvic acid 50 in water as solvent was successfully used for obtaining the 2-aryl-quinoline-4-carboxylic acids 51 (Scheme 15).^102^ However, classical acid reaction is still used for bioactive 2-phenyl-quinoline-4-carboxylic acid derivatives.^103^

**Scheme 16:**
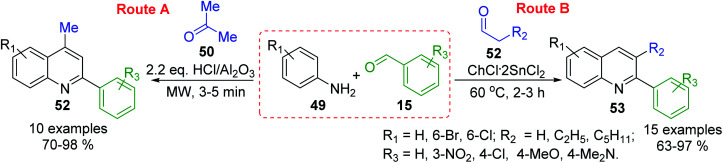
Type ABC three component Doebner–Miller reactions for the preparation of substituted 2-arylquinolines.

MW-assisted solvent-free 3-C Doebner–Miller reaction of arylamines 49, benzaldehydes 15 and active methylene compounds (*e.g.*, acetone 50) in the presence of HCl/Al_2_O_3_ catalyst allowed the preparation of potentially bioactive substituted 4-methyl-2-phenylquinolines (lepidine derivatives) 52 in good yields (Scheme 16, route A),^104^ while related reaction using condensation between similar anilines and benzaldehydes, and enolizable aldehydes 53 in choline chloride/tin(ii) chloride (ChCl·2SnCl_2_, 1 : 2) as a deep eutectic solvent conducted to the formation of diverse 2-aryl-3-alkylquinolines 54^105^ (Scheme 16, route B). These latter quinoline molecules can also be efficiently prepared in the presence of butylpyridinium tetrachloroindate-(III), [bpy][InCl_4_], ionic liquid catalyst and solvent^106^ or from respective aldimines and enolizable aldehydes using 1 mol% of iodine as catalyst in benzene at reflux at 0.5 h.^107^

Noteworthy, that three basic concepts of green chemistry (atom economy, safer solvents and design for energy efficiency) are included in both procedures (methods A and B), which can be considered as modified Miller–Povarov reactions.^108^ Moreover, these multi-component reactions (MCRs) are very important for antiparasitic medicinal chemistry research because they can generate functional group diversity. The easy availability of starting materials, and the rapid generation of product diversity by means of multicomponent operation are their advantages. Another type of MCRs for simple substituted 2-arylquinolines involves the condensation of benzaldehydes, anilines and nitroalkanes (nitroethane, nitropropane and nitrobutane) in the presence of catalytic amount of Fe(iii) chloride.^109^ This one-pot, three-component domino strategy works under ambient air and affords the quinoline products in high yields.

**Scheme 17:**
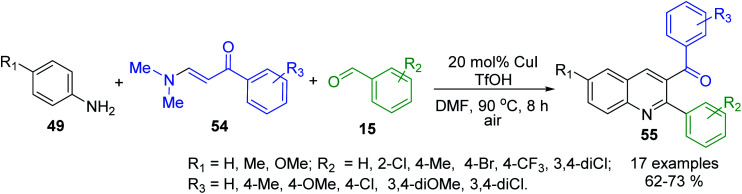
Synthesis of 3-acyl-2-arylquinolines *via* the enaminone modified Povarov reaction.

The selective synthesis of 3-acyl-2-arylquinolines 55 by utilizing anilines 49, benzaldehydes 15 and *N*,*N*-dimethyl enaminones 54 as useful alkene surrogates in the presence of TfOH and CuI (20 mol%) in DMF belongs to these multi-component reactions^110^ (Scheme 17). It is believed that under acidic conditions transamination of between the enaminone 54 and aniline 49 takes place first to provide NH-aryl-enaminone derivative that reacts then with aldehyde 15 to give iminium cation those decomposition leads to the formation of enaminone and aldimine based on 49 and 15. Then, the subsequent nucleophilic addition of enaminone 54 to formed aldimine gives ionic intermediate, which provides *N*,*N*-dimethylamino tetrahydroquinoline after the cyclization. Finally, the elimination of HNMe_2_ and the following aromatization of tetrahydroquinoline ring furnish the products.

**Scheme 18:**
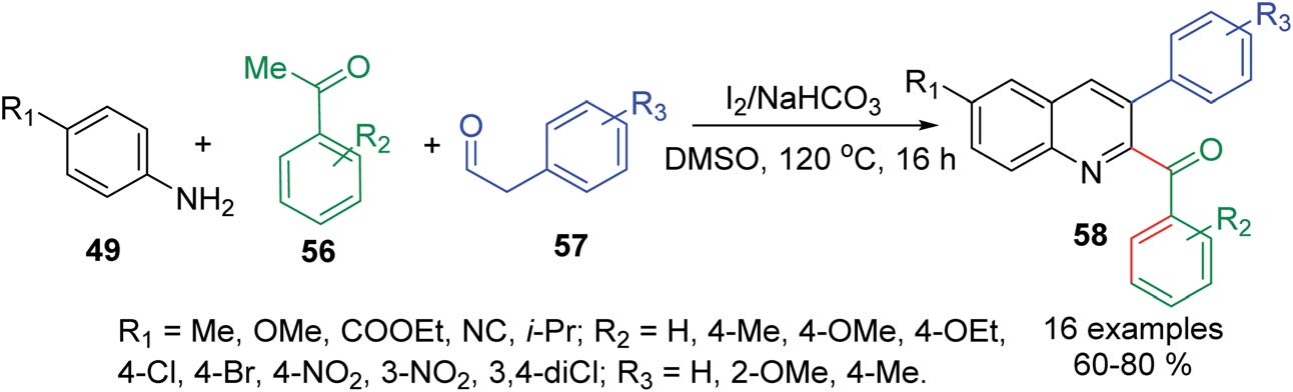
Synthesis of 2-acyl-3-arylquinolines through I_2_/amine promoted Povarov-type reaction.

Similar Povarov-type reaction of arylamines 49, acetophenones 56 and arylacetaldehydes 57 as alkene alternates with I_2_ as mild acid catalyst in DMSO at 120 °C affords 2-acyl-3-arylquinolines 58 in moderate to high yields (Scheme 18).^111^ In this case, while acetophenone 56 is converted into respective arylglyoxal that reacts with 49 to give keto-aldimine, another molecule of aniline 49 and arylacetaldehyde 57 produce respective imine, which was then rapidly converted into enamine intermediate. Then its subsequent interaction with keto-aldimine *via* a formal I_2_-catalysed [4 + 2]-cycloaddition allows obtaining quinoline product.

**Fig. 6:**
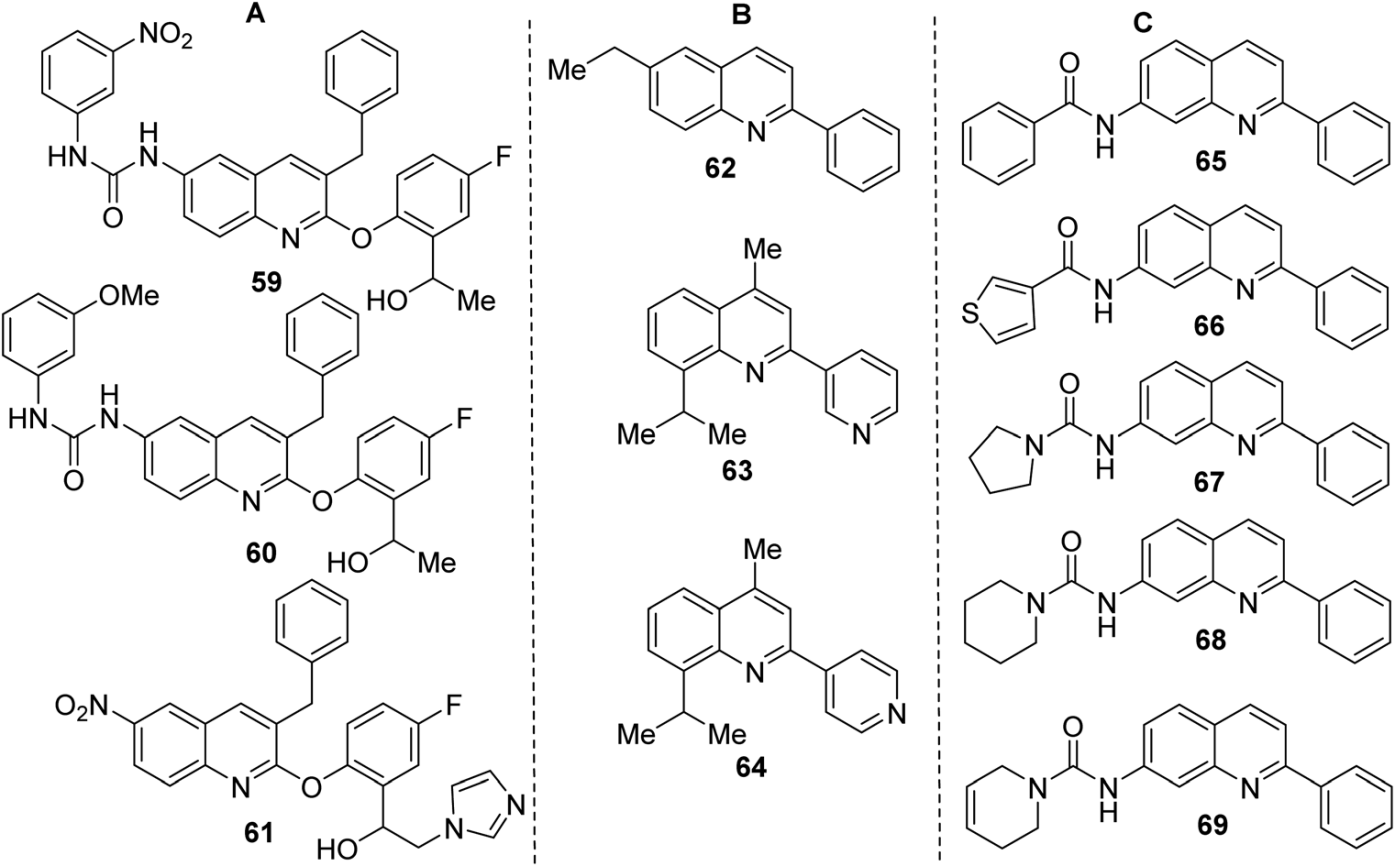
Structures of promising antiprotozoal quinoline agents.

Like 2-aryloxy-3-benzylquinolines 59–61 (Fig. 6A), both final products 55, 58 are interesting and useful models in biological research. Quinolines 59–61 demonstrated excellent *in vitro* antitrypanosomal (IC_50_ = 0.25–2.0 μM, *T. cruzi*) and antileishmanial activity (IC_50_ = 1.7–3.1 μM, *L. infantum*), which is superior to frontline drugs benznidazole (IC_50_ = 3.66 μM), nifurtimox (IC_50_ = 1.8 μM) and comparable to amphotericin B (IC_50_ = 0.25 μM).^112^ Analogous 3-diaryl ether quinoline derivatives were shown to possess growth inhibitory activity to *Toxoplasma gondii* and proved non-cytotoxic to the host cells.^113^ Quinoline 59 with nitro urea function showed the most activity against both *Trypanosoma* spp. (IC_50_ = 0.25–1.0 μM, *T. cruzi* and *T. brucei brucei*) and *L. infantum* (IC_50_ = 1.70 μM). Interestingly, 2-phenylquinoline 62 (Fig. 6B), another alkaloid found in *G. longiflora* extracts, is inactive in antileishmanial tests (IC_50_ > 100 μM).

However, its derivatives and analogues exhibited interesting antitrypanosomal properties that stimulate further investigation on this theme. For example, the 6-ethyl-2-phenylquinoline 63 displayed a good effect on promastigotes and intracellular amastigotes of *L. braziliensis* parasites (IC_50_ < 50 μM; EC_50_ 6.0 μM) without disturbing the viability of the host cells,^114^ while the 3-pyridinylquinoline 64 showed *in vitro* response against both live forms of *T. cruzi* and *L. chagasi* parasites and was nontoxic on mammalian cells.^115^

However, the most interesting molecules based on a simple 2-arylquinoline skeleton resulted be 7-amino-2-phenylquinoline derivatives 65–69 (Fig. 6C), which were more active than the reference drug (benznidazole) *in vitro* screens against bloodstream forms of *T. cruzi*.^116^ They displayed EC_50_ < 3 μM (<4-fold than benznidazole). Among them, quinolines 67 and 69 were tested *in vivo* models. The first molecule showed promising results in mice with *T. cruzi* and *T. brucei* infection, reaching 70% reduction of the parasitemia load and it cured 2 of 4 mice, while the second was active in mice with *T. brucei* infection curing all 4 mice (100% cure rate). The synthesis of key 7-amino-2-phenylquinoline precursor was achieved in 3 steps through the Friedländer reaction starting with 4-bromo-2-nitrobenzaldehyde.^116^ In this context, it should be commented that there are still few efficient, direct synthesis for C-4 unsubstituted 2-arylquinolines using 3-C Povarov reaction of anilines, aromatic aldehydes and rich electron alkenes (*e.g.*, alkyl vinyl ethers or *N*-vinyl amides).^117–120^ that could be a good alternative route to quinolines 65–69.

**Scheme 19:**
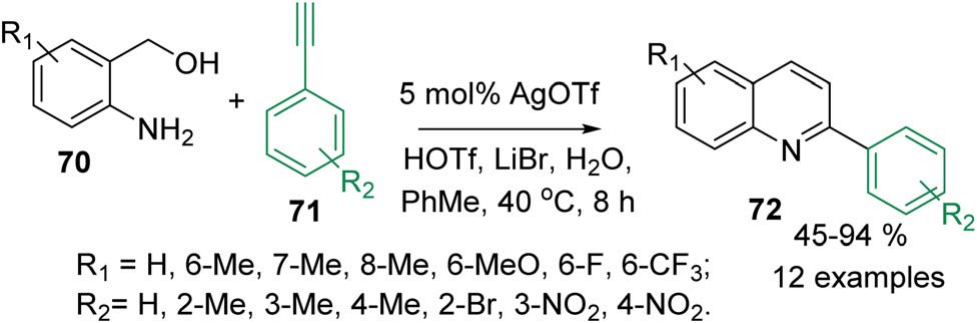
Synthesis of 2-arylquinoline *via* a modified Friedländer reaction of 2-aminobenzyl alcohols and arylacetilenes.

Several extensions to Friedländer synthesis can provide also C-4 unsubstituted 2-arylquinolines. For example, AgOTf/HOTf-catalysed reaction of 2-aminobenzyl alcohols 70 and arylacetilenes 71 in the presence of LiBr and H_2_O in toluene in air afforded desired 2-arylquinolines 72 in moderate to excellent yields (Scheme 19).^121^ Very recently, synthesis of analogous quinolines *via* nickel-catalysed dehydrogenative coupling of 70 and methyl ketones 56 in boiling toluene was reported by De Sarkar and colleagues.^122^

It should be noted that early Patil and Raut developed a copper-catalysed tandem addition/cycloisomerisation reaction giving access to 2-arylquinolines using 2-aminobenzaldehydes and terminal alkynes as substrate^123^ and a microwave-assisted solid acid-catalysed synthesis of 2-arylquinolines from anilines and cinnamaldehydes in the presence of montmorillonite K-10 was also reported by Török and co-workers.^124^ Recently, Liang and co-workers reported a simple method for the synthesis of substituted quinoline derivatives by copper-catalysed annulation of anilines with terminal acetylene esters, *e.g.*, 1-phenylprop-2-ynyl acetate^125^ and Guo and colleagues^126^ developed an aerobic oxidative protocol based on a Cu-catalysed C–H cyclization (10 mol% CuCl_2_·2H_2_O) of simple anilines with acetophenones and DMSO as a one-carbon source for preparing easily diverse 2-arylquinolines. Instead of 2-aminobenzyl alcohols, 2-nitrobenzyl alcohols can also be used in the 2-arylquinoline synthesis that is catalysed by Fe-catalyst and formic acid as a redox moderator to fill the electron gap of the global redox condensation process.^127^

**Scheme 20:**
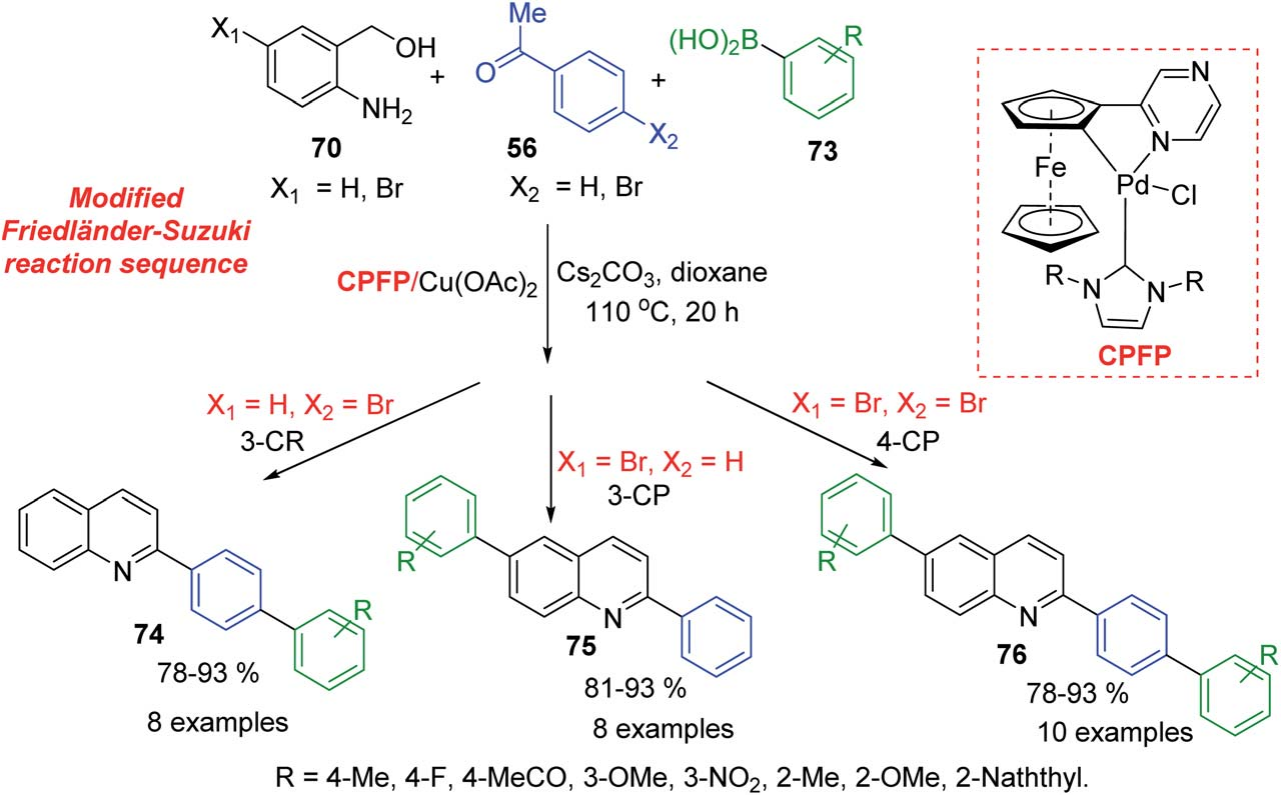
Modular synthesis of aryl quinolines *via* modified Friedländer–Suzuki reaction sequence.

Because 2-aminobenzyl alcohols are more inexpensive and stable than 2-aminobenzaldehydes, which were used in Friedländer reaction, development of catalysed process for C-4 unsubstituted 2-arylquinolines is important topic of modern organic chemistry. Xu and colleagues developed a NHC-modulated Pd/Cu-cocatalysed three (four)-component reaction of aminobenzyl alcohols 70, acetophenones 56, and arylboronic acids 73 in air. This interesting protocol is based on modified Friedländer–Suzuki reaction sequence and provides an efficient access to a variety of aryl substituted quinolines 74–76 (Scheme 20).^128^ Obtained quinolines are both interesting biologically active molecules for antiparasitic screening and suitable ligands for the preparation of phosphorescent complexes.

**Scheme 21:**
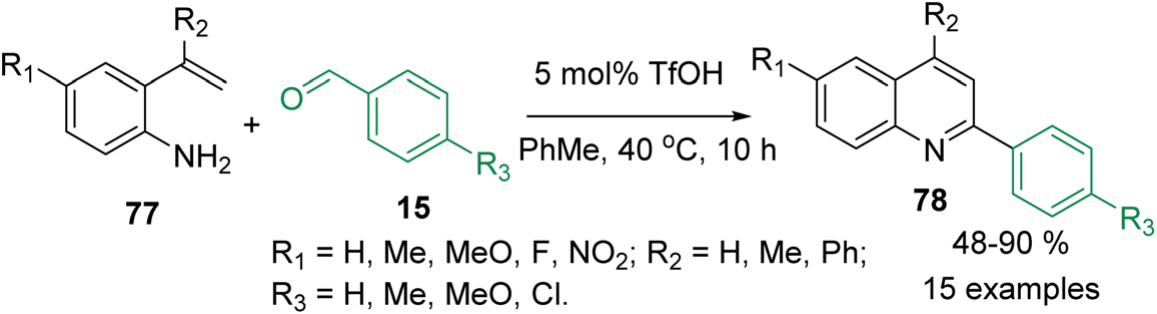
Brønsted acid-mediated reactions of 2-vinylaniline with aldehydes to give 2-arylquinolines.

The 2-vinylanilines 77 readily react with benzaldehydes 15 in the presence of triflic acid, TfOH (5.0 mmol%) in warm toluene to afford also C-4 unsubstituted 2-arylquinolines 78 in moderate to excellent yields (Scheme 21).^129^ It was assumed that first, the arylamine and the benzaldehyde are easily condensed to yield the imine product, followed by the attack of the double bond to the protonated imine, and then rearrangement occurs, which is followed by deprotonation and oxidation to furnish the quinoline product.

**Scheme 22:**
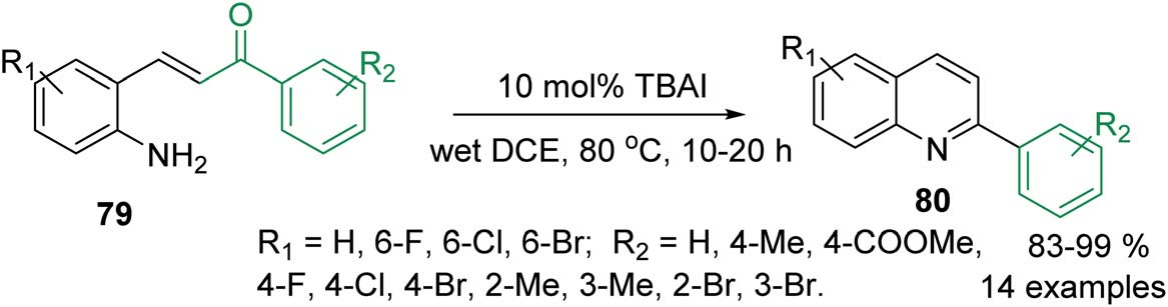
An efficient iodine-promoted intramolecular condensation of 2-aminostyryl ketones into 2-arylquinolines.

Another approach to the synthesis of C-4 unsubstituted 2-arylquinolines is the iodine-catalysed intramolecular condensation of 2-aminostyryl ketones 79 using tetrabutylammonium iodide (TBAI) as a nucleophilic catalyst in dichloroethane (DCE) (Scheme 22).^130^ The formation of quinolines 80 occurred through the conjugate addition of iodide to 79, furnishing the corresponding β-iodoketones, which could adopt the *s-cis* conformation and the amino and carbonyl groups can react (*via* free rotation about the Cα–Cβ single bond) to their condensation followed by the elimination of hydrogen iodide giving the corresponding 2-arylquinolines. The same authors showed that benzylamine works well in this cyclisation.^131^

This simple operation looks very attractive; excellent yields, broad substrate scope, no need of any special equipment. However, 2-aminostyryl ketones 79 are not commercially available and its preparation from 2-nitrobenzaldehydes through aldol condensation of methyl ketone derivatives with followed by the reduction of a nitro group into an amino group may be laborious and expensive comparing with the 3-C Povarov reaction. Direct C–H arylation reaction of *N*-methoxy-4-methylquinoline-1-ium tetrafluoroborate salts and arylboronic acids in the presence of 10 mol% AgNO_3_ and 1 equiv. Na_2_S_2_O_8_ in DCM : H_2_O (1 : 1) at 25 °C under air can give smoothly 2-arylquinolines.^132^

**Scheme 23:**
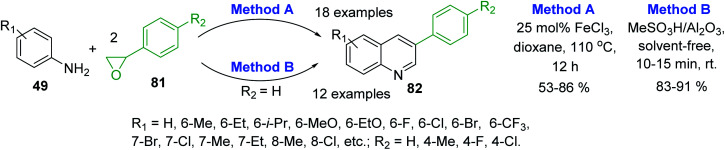
Synthesis of 3-arylquinolines from anilines and styrene oxide using different protocols.

Recent syntheses of 3-arylquinoline molecules 82 consist in the one-pot reaction of anilines 49 and styrene oxide 81. Wang and co-workers proposed to use an inexpensive FeCl_3_ as promoter for this reaction that occurred well in 1,4-dioxane at 110 °C for 12 h (Scheme 23, method A).^133^ In 2017, a new procedure for these quinolines was reported by Sharghi and co-workers. They developed one-pot reaction of anilines and styrene oxide under solvent-free conditions in the presence of Al_2_O_3_/MeSO_3_H, at room temperature (Scheme 23, method B).^134^ The use of this catalytic system allowed performing efficiently the synthesis under very short reaction time (10–15 min) and room temperature in solvent-free manner.

Diverse 3-arylquinolines can be also available from anilines and terminal acetylenes, their simple synthesis involves the use of DMSO as both the solvent and the C1 source and Cp*Co(CO)I_2_, AgNTf_2_, and HOPiv as the catalytic system.^135^

**Scheme 24:**
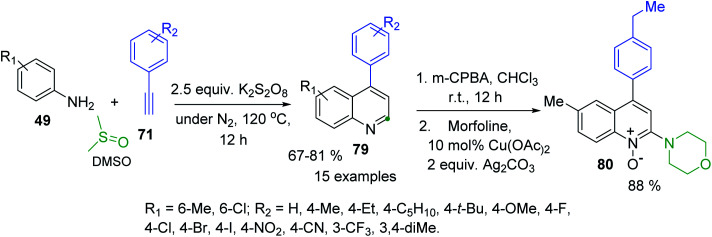
Synthesis of 4-arylquinolines and its C–H functionalization *via N*-oxides.

4-Arylquinolines were not easily available as their C-2-aryl analogues. Some 6(7)-(di)substituted 4-phenylquinolines were obtained in good yields *via* a modified Doebner reaction of anilines and phenylacrolein in the presence of AgOTf (5 mol%) and the additive TfOH (5 mol%) in toluene under atmospheric conditions.^136^ But recently, diverse simple procedures based on catalysed C–H functionalization strategy have been developed.^137–139^ These protocols involve metal-Co(iii), Co(iii)/Ag(i), Fe(iii) catalysis in the 3-C reaction of anilines, aryl methyl ketones (or arylacetilenes) and paraformaldehyde (or DMSO) as C_1_ building block of quinoline products. Although all these transition metal catalysed approaches allow a convenient access to 4-arylquinolines, the use of expensive and toxic transition metal catalysts and additives are major drawbacks associated with them. Considering these problems, Tiwari and colleagues developed an atom economical and transition metal free synthesis of 4-arylquinolines 83 from readily available from anilines 49 and arylacetilenes 71 in the presence of K_2_S_2_O_8_ and DMSO (Scheme 24).^140^ It should be noted the synthetic utility of this methodology was further extended to the synthesis of medicinally important 4-aryl-2-morpholinoquinolines like 84 through a formation of the respective quinoline *N*-oxides, which can be used in direct C–H alkenylation reaction (see, Scheme 14).

**Scheme 25:**
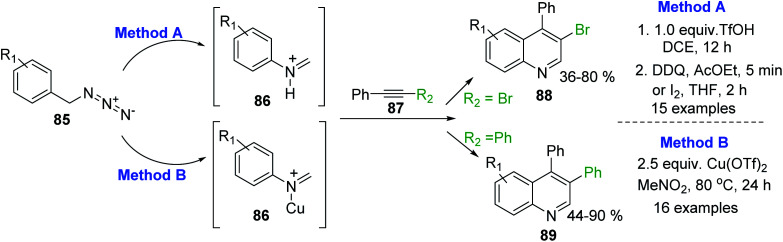
Synthesis of 3-substituted 4-aryquinolines using benzylic azides and internal alkynes.

An efficient CH_3_SO_3_H-promoted synthesis of 4-arylquinolines from readily available anilines, acetophenones and DMSO under air was also reported.^141^ Synthetic procedures for analogous 3,4-disubstituted quinolines 88,89 through the intermolecular cyclization reaction of arylmethyl azides 85 and internal alkynes 87 have been recently described.^142–144^ This transformation is based on an acid-promoted Aubé-Schmidt's rearrangement of benzylic azides 85 giving iminium ion intermediate 86,^145^ which can be efficiently trapped by internal alkynes 87. Tummatorn and his team developed two-step nucleophilic addition–anullation (TfOH–DCE)/oxidation (DDQ–AcOEt or I_2_–THF) sequence protocol that allowed to obtain 4-aryl-3-bromoquinolines 88 in moderate to good yields^143^ (Scheme 25, method A), while Cheng and co-workers proposed to use Cu(OTf)_2_ as both a Lewis acid and oxidant.^144^ Reactions of azides 85 and diphenylacetylene 87 (R_2_ = Ph) in MeNO_2_ at 80 °C gave new 3,4-diphenylquinolines 89 in good to excellent yields (Scheme 25, method B).

An interesting synthesis of potential bioactive 4-aryl-2-(quinolinyl-2)-quinolines through Cu-catalysed cascade oxidative cyclization reaction of 2-(1-phenylvinyl)anilines and 2-methylquinolines has been reported by Yan and colleagues in 2017.^146^

Remarkably, since 2008 there has been an extraordinary increment in the number of reported procedures describing the construction of quinolines that have a variety of functional groups at different positions, especially 2,4-diphenylquinolines. These quinoline derivatives are very important and promising molecules for the synthesis of OLED materials, crucial ligands in the formation of other conjugated molecules with enhanced optoelectronic properties. But, they could also be interesting potentially antiparasitic agents. The main methods for their preparation are based on Skraup–Doebner–Miller, Friedländer, Combes–Conrad–Limpach condensations and Povarov reaction. All these provide adequate diversity and substitution for the structural decoration of the quinoline ring. There are numerous procedures using transition metal-catalysed of arylamines and 1,3-dicarbonyl compounds or ene carbonyl/acetylenic carbonyl compounds.

The most popular metal catalysts used in the extensions of these traditional quinoline syntheses are iron, gold, silver, and copper.^147–152^

**Scheme 26:**
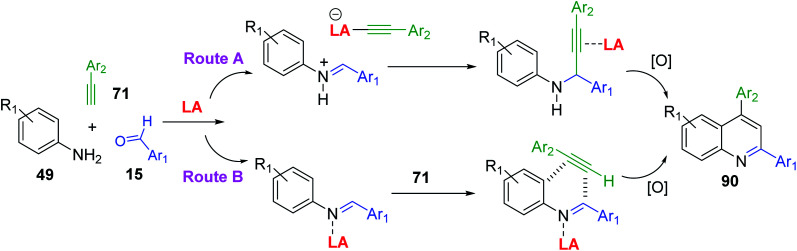
Pathways for the A^3^ Povarov reactions.

A^3^-type Povarov reactions of primary aromatic amine 49, aldehyde 15 and alkyne 71 (A^3^ synthesis) to give 2,4-diphenylquinolines 90 (Scheme 26) have become very popular in these recent years can provide a good functional group diversity. These transformations were performed in the presence of Cu(OTf)_2_ (5 mol%)^153^ or Zn(OTf)_2_ (5 mol%)^154^ without any solvents. Molecular iodine in MeNO_2_ and borane tris(pentafluorophenyl)borane (B(C_6_F_5_)_3_) in “wet” DCE were also employed in these acid-catalysed A^3^ Povarov reactions.^155,156^ It is believed that in the LA-catalysed transformation an aldimine, produced *in situ*, undergoes nucleophilic attack by an alkyne to yield a propargylamine. The *N*-aryl propargylamine then reacts further, forming a quinoline motif upon cyclization/oxidation. This is so-called alkyne activation route (Scheme 26, route A). Another possible pathway could proceed with Brønsted and certain Lewis acids, which instead activate the imine to nucleophilic attack by the alkyne, in a variant of the Povarov reaction (Scheme 26, route B).

The solvent-free synthesis of 2,4-diphenylquinolines 90*via* A^3^ Povarov reaction of 49, 15 and 71 in the presence of FeCl_3_ at room temperature was also reported using mechano-chemical ball milling technique. With this protocol, seventeen 2,4-diphenylquinolines were easily prepared in good to excellent yield (74–95%) just by washing the resulting reaction mixture with water and recrystallizing in EtOH/H_2_O without any extraction or column chromatography work-up.^157^ Recently, the triazole gold (TAAu)-catalysed A^3^ coupling reaction to synthesize similar quinoline derivatives 90 was developed.^158^

**Scheme 27:**
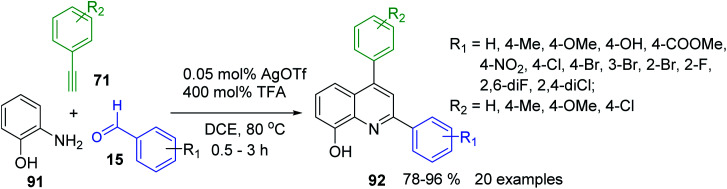
Synthesis of 8-hydroxyquinoline derivatives from aminophenol, aldehydes and alkynes.

A^3^-type Povarov reaction resulted also in very suitable tool for the construction of 2,6-diaryl-8-hydroxyquinolines 92, important scaffolds in pharmacology, medicinal chemistry^159,160^ and functional materials chemistry.^161^ Their synthesis was possible under cooperative AgOTf/TFA catalysis with commercially available materials 15, 71 and 91 (Scheme 27).^162^

A CuCl and AuCl dual catalysed A^3^-type Povarov cascade reaction of anilines, various substituted benzaldehydes and hydroxyalkyl substituted alkynes, which provides efficiently 4-hydroxyalkyl-2-arylquinoline derivatives with high yields, was reported by Lin and colleagues.^163^ Recently, some interesting Povarov reaction variants, which involve Lewis acid- and Brønsted acid-catalysed dehydrogenative [4 + 2] cycloaddition of *N*-benzyl anilines (which covert into aldimines or their *N*-oxides) and alkenes (alkynes) under oxidant reaction conditions to give diverse 2,4-diphenylquinolines have been reported.^164–166^ Having a reach library of polyfunctionalized 2,4-arylquinoline molecules, it is strange that there are no reports about their antileishmanial and trypanocidal properties until nowadays.

## Conclusion

Interest in the synthesis and chemistry of quinoline derivatives is not lost in our modern times, on the contrary, new methods and protocols for new quinoline molecules are constantly developing.^167,168^ Always looking for more versatile and sustainable protocols, this development is now strongly related to the implementation of the concepts of green chemistry. Today, green construction of these heterocyclic molecules is the responsibility of organic chemists. It's nice to see that practically all the principles of green chemistry are involved in the current synthesis of quinolines. There are known many types of different, efficient MW-assisted, metal-free, solvent-free, multi-component quinoline synthesis, *etc.* in the modern literature. However, considering great biological significance of quinoline molecules as antiparasitic agents useful in the treatments of Chagas disease, African sleeping sickness and leishmaniasis, the success in the development of synthetic methods for new antiparasitic quinolines is opaque, less impressive. Today, new drugs for these neglected tropical diseases are still urgently needed.

In this respect, simple synthetic quinolines could be suitable and promising models for developing new, more effective candidates, which contributes to reasonable chemotherapies for these devastating diseases. Nevertheless, the development and advances of new agents based on the quinoline skeleton against these diseases are not the same: while several very promising molecules against *Leishmania* parasites are discovered and some of them are under clinic studies, search for quinoline molecules against *T. cruzi* and *T. brucei* protozoa is less successful. Having an enormous and powerful synthetic arsenal to synthesize polyfunctionalized quinoline derivatives, we would like to hope that these modern, green synthetic methodologies will result in developing more specific antitrypanosomal drug candidates in the near future.

## Conflicts of interest

The authors confirm that this article content has no conflict of interest.

## Supplementary Material
